# DNMT3B overexpression contributes to aberrant DNA methylation and MYC-driven tumor maintenance in T-ALL and Burkitt’s lymphoma

**DOI:** 10.18632/oncotarget.20176

**Published:** 2017-08-10

**Authors:** Candace J. Poole, Wenli Zheng, Atul Lodh, Aleksey Yevtodiyenko, Daniel Liefwalker, Honglin Li, Dean W. Felsher, Jan van Riggelen

**Affiliations:** ^1^ Augusta University, Department of Biochemistry and Molecular Biology, Augusta, GA 30912, USA; ^2^ Stanford University School of Medicine, Division of Oncology, Departments of Medicine and Pathology, Stanford, CA 94305, USA

**Keywords:** MYC, DNMT3B, DNA methylation, leukemia/lymphoma

## Abstract

Aberrant DNA methylation is a hallmark of cancer. However, our understanding of how tumor cell-specific DNA methylation patterns are established and maintained is limited. Here, we report that in T-cell acute lymphoblastic leukemia (T-ALL) and Burkitt’s lymphoma the *MYC* oncogene causes overexpression of DNA methyltransferase (DNMT) 1 and 3B, which contributes to tumor maintenance. By utilizing a tetracycline-regulated *MYC* transgene in a mouse T-ALL (EμSRα-tTA;tet-o-MYC) and human Burkitt’s lymphoma (P493-6) model, we demonstrated that DNMT1 and DNMT3B expression depend on high MYC levels, and that their transcription decreased upon MYC-inactivation. Chromatin immunoprecipitation indicated that MYC binds to the *DNMT1* and *DNMT3B* promoters, implicating a direct transcriptional regulation. Hence, shRNA-mediated knock-down of endogenous MYC in human T-ALL and Burkitt’s lymphoma cell lines downregulated DNMT3B expression. Knock-down and pharmacologic inhibition of DNMT3B in T-ALL reduced cell proliferation associated with genome-wide changes in DNA methylation, indicating a tumor promoter function during tumor maintenance. We provide novel evidence that MYC directly deregulates the expression of both *de novo* and maintenance DNMTs, showing that MYC controls DNA methylation in a genome-wide fashion. Our finding that a coordinated interplay between the components of the DNA methylating machinery contributes to MYC-driven tumor maintenance highlights the potential of specific DNMTs for targeted therapies.

## INTRODUCTION

*c-MYC* (here referred to as *MYC*) encodes for a transcription factor that is a key regulator of a wide variety of cellular processes (reviewed in [1]). The diverse functions of MYC depend on its ability to control the expression of a large number of genes (reviewed in [2]). While in most non-malignant cells MYC levels are low, its constitutive expression is linked to the pathogenesis of many human cancers. In particular, MYC is associated with hematopoietic malignancies such as Burkitt’s lymphoma and T-cell acute lymphoblastic leukemia (T-ALL) (reviewed in [3, 4]). MYC exhibits its neoplastic properties by causing autonomous cellular proliferation and growth, blocking differentiation, and genomic destabilization (reviewed in [5]). The finding that tumors can be dependent on MYC expression (oncogene addiction), and can undergo sustained regression upon inactivation of the oncogene [6], holds exceptional promise for targeting MYC itself or its network for therapeutic purposes. However, despite extensive efforts in this regard, neither a specific pharmacologic inhibitor has been successfully translated into the clinical setting, nor the exact mechanism of how elevated MYC levels reprogram cells to promote cancer is known.

MYC is well known as a site-specific transcription factor regulating the expression of hundreds of target genes. MYC forms heterodimers with MAX, binding to a DNA-motif termed E-box (CACGTG) [7]. MYC–MAX then recruits histone acetyltransferases (HATs) like GCN5 and TIP60, increasing the acetylation of histones in the vicinity of its binding site, thereby trans-activating canonical target genes [8, 9]. However, in addition to its function as an activator, MYC can also repress transcription of genes through interactions with other transcription factors such as SP1 and MIZ-1 (reviewed in [10]). In complex with MIZ-1, MYC–MAX trans-repress growth-inhibitory genes including a number of cycline-dependent kinase inhibitors [11]. While numerous MYC target genes have been identified in various tumor types, the lack of overlap makes it difficult to assign MYC’s oncogenic properties to a particular set of genes, suggesting alternative mechanisms.

Hence, the classic model has recently been extended to incorporate MYC’s role as a global regulator of transcription. There is growing evidence that MYC induces genome-wide changes in the epigenetic landscape of the cell as an important part of its neoplastic features, including alterations of post-translational histone modifications (reviewed in [12]). N-MYC-induced genome-wide acetylation of histones results in part from transcriptional activation of the histone acetyltransferase, *GCN5* [13]. By controlling transcription of *GCN5*, MYC exerts broad effects on global chromatin structure establishing a tumor-cell specific pattern. In parallel, MYC suppresses chromatin regulators including *SIN3B*, *HBP1*, *SUV420H1*, and *BTG1* through *miR-17-92* [14]. Correspondingly, we reported that the inactivation of MYC in T-ALL causes changes in global histone H4 acetylation, H3K9me3 and H3K4me3 associated with cellular senescence and tumor regression [15]. These results suggest that in tumors that elicit oncogene addiction, the *MYC* oncogene establishes and maintains a genome-wide epigenetic state, while MYC inactivation triggers dramatic alterations in chromatin structure leading to cellular senescence as an important mechanism of tumor regression. However, how DNA methylation contributes to MYC-driven tumor maintenance, and how genome-wide aberrant DNA methylation patterns are established and maintained remains largely unknown.

DNA methylation represents an important feature that controls gene expression programs in mammalian development and disease [16, 17]. DNA methylation is established and maintained by the interplay between three enzymatically active DNA methyltransferases (DNMT1, DNMT3A, and DNMT3B) (reviewed in [18]). DNMT1 is considered to be responsible for maintenance of DNA methylation, whereas DNMT3A and DNMT3B are *de novo* methyltransferases that establish new DNA methylation patterns [16]. The current paradigm places the majority of DNA methylation in non-malignant cells at centromeric sequences and transposable elements to maintain genomic stability. Alterations of DNA methylation patterns by improper *de novo* methylation is a common event in human neoplasia and is known to contribute to tumorigenesis [19–21]. Tumor cells typically display global hypomethylation of repetitive DNA elements which contributes to genomic instability, while promoter and CpG island hypermethylation extinguishes transcription of tumor suppressor genes.

Together, these observations suggest that oncogenes, such as *MYC*, establish and maintain tumor-cell specific DNA methylation patterns on a genome-wide level through modulating individual components of the DNA methylating machinery. Here, we report that in T-ALL and Burkitt’s lymphoma, oncogenic MYC deregulates the expression of both DNMT1 and DNMT3B, which in turn establishes and maintains tumor cell-specific DNA methylation patterns in a genome-wide fashion with importance for tumor maintenance

## RESULTS

### DNMT1 and DNMT3B are overexpressed in T-ALL and Burkitt’s lymphoma cell lines

MYC-driven hematopoietic malignancies harbor aberrant DNA methylation patterns. However, how these tumor cell-specific marks are established and maintained through the interplay between the DNA methylating machinery is still elusive. To unravel the role of DNA methyltransferases (DNMTs) in MYC-driven hematopoietic malignancies, we performed expression profiling for DNMT1, DNMT3A and DNMT3B comparing MYC-driven T-ALL and Burkitt’s lymphoma cells to non-malignant control cells.

First, we took advantage of EμSRα-tTA;tet-o-MYC transgenic mice, in which the ectopic expression of MYC in hematopoietic lineages gives rise to T-ALL [6]. The malignant phenotype is characterized by enlarged thymus, lymph nodes and spleen harboring CD4+ CD8+ double positive T-lymphocytes resembling the human disease [6]. We previously reported that T-ALL in this model elicits oncogene addiction, and that MYC inactivation causes tumor regression by triggering cellular senescence [15, 22]. Tumor regression was associated with global epigenetic changes, highlighting the notion that MYC controls chromatin organization in a genome-wide fashion.

**Figure 1:**
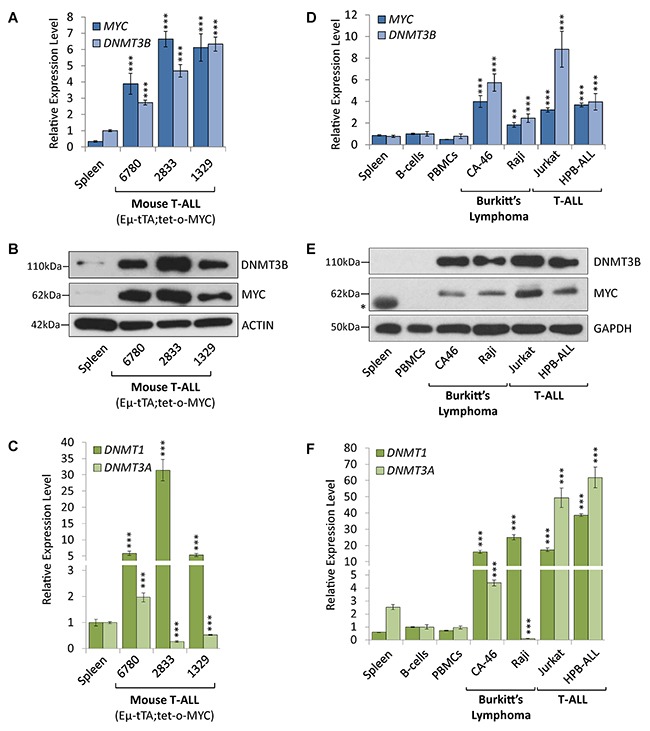
DNMT1 and DNMT3B are overexpressed in mouse and human T-ALL and Burkitt’s lymphoma cell lines Expression analysis of DNMT1, DNMT3A and DNMT3B in MYC-driven T-ALL and Burkitt’s lymphoma compared to non-malignant tissue. **(A)** RT-qPCR of *MYC* and *DNMT3B*, **(B)** Western blot analysis of MYC and DNMT3B, and **(C)** RT-qPCR of *DNMT1* and *DNMT3A* in cell lines (6780, 2833 and 1329) derived from a transgenic T-ALL mouse model (EμSRα-tTA;tet-o-MYC) in comparison to spleen tissue obtained from wild-type C57BL/6J mice. **(D)** RT-qPCR of *MYC* and *DNMT3B*, **(E)** Western blot analysis of MYC (* marks a non-specific band) and DNMT3B, and **(F)** RT-qPCR of *DNMT1* and *DNMT3A* in human T-ALL (Jurkat and HPB-ALL) and Burkitt’s lymphoma (CA46 and Raji) compared to normal spleen, peripheral blood mononuclear cells (PBMCs) and B-cells obtained from healthy donors. RT-qPCR data was normalized to *UBC* or *RPL13A*; ACTIN or GAPDH serves as loading control for Western blot. Error bars represent mean ± SEM; *n* = 3; two-tailed Student’s *t*-test: **P < 0.01; ***P < 0.001.

RT-qPCR and Western blot analysis of tumor cells derived from EμSRα-tTA;tet-o-MYC transgenic mice indicate that DNMT3B expression levels were elevated in MYC-driven T-ALL compared to normal spleen from wild-type C57BL/6J mice (Figure 1A, 1B, 1C and Supplementary Figure 1). T-ALL cells (6780, 2833 and 1329) expressed significantly increased *DNMT3B* mRNA (2.73-, 4.68- and 6.33-fold, respectively, *P*<0.001) and corresponding protein levels than non-malignant tissue. We also found *DNMT1* mRNA to be overexpressed in all three T-ALL samples (5.79-, 31.41- and 5.28-fold, *P*<0.001, respectively). In contrast, *DNMT3A* mRNA levels were lower in 2833 and 1329 (-3.68- and -1.87-fold, respectively, *P*<0.001) than in non-malignant tissue; only 6780 expressed more DNMT3A (1.97-fold, *P*<0.001). As expected, MYC expression levels were very low in non-malignant tissues compared to tumor cells.

Next, we compared a panel of human T-ALL and Burkitt’s lymphoma cell lines to non-malignant human spleen, peripheral blood mononuclear cells (PBMCs) and B-cells obtained from healthy donors (Figure 1D, 1E and 1F). Both T-ALL (Jurkat and HPB-ALL) and Burkitt’s lymphoma (CA46 and Raji) cell lines showed significantly increased *DNMT3B* mRNA (3.18- to 8.82-fold, respectively, *P*<0.001) and protein expression in comparison to non-malignant spleen and B-cells, respectively. Similarly, *DNMT1* mRNA expression was increased in all human T-ALL and Burkitt’s lymphoma cell lines (17.30- to 41.54-fold, *P*<0.001) compared to normal spleen and B-cells, respectively. In contrast, *DNMT3A* mRNA was increased in all human T-ALL (49.35- and 61.88-fold, *P*<0.001), but not in all Burkitt’s lymphoma cell lines. While we detected 1.73-fold (*P*<0.001) more *DNMT3A* in CA46 cells, Raji cells showed a significantly decreased -27.91-fold (*P*<0.001) *DNMT3A* mRNA expression.

Based on our expression profiling, we conclude that DNMT1 and DNMT3B are consistently overexpressed in MYC-driven T-ALL and Burkitt’s lymphoma cell lines, while DNMT3A is overexpressed in human T-ALL cell lines only.

### DNMT1, DNMT3A and DNMT3B are overexpressed in clinical T-ALL and Burkitt’s lymphoma specimens

**Figure 2:**
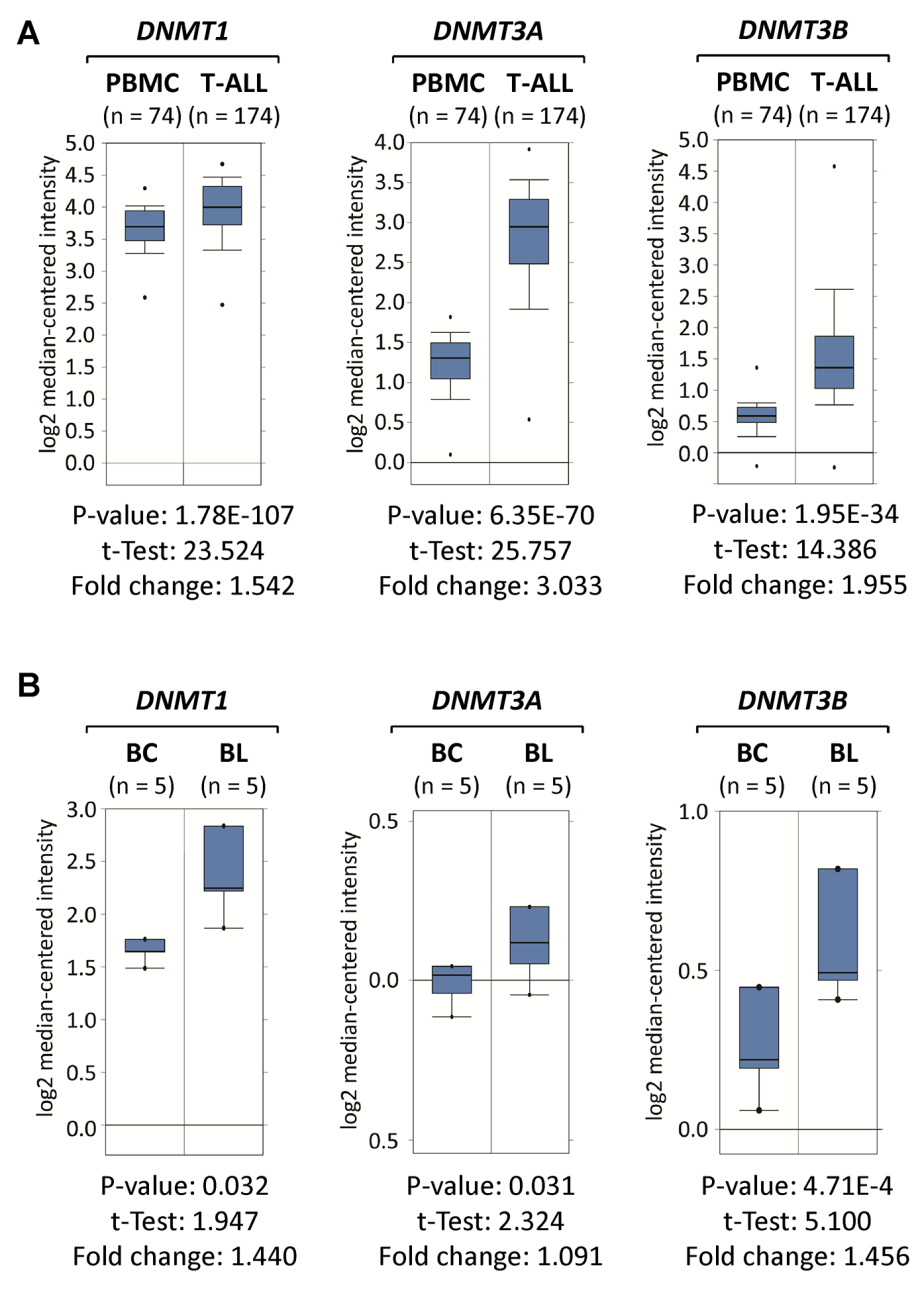
DNMT1, DNMT3A and DNMT3B are overexpressed in clinical T-ALL and Burkitt’s lymphoma specimens *DNMT1*, *DNMT3A* and *DNMT3B* expression profiles in clinical specimens obtained from Oncomine (http://www.oncomine.org) are displayed in comparison to non-malignant control cells. **(A)** *DNMT1*, *DNMT3A* and *DNMT3B* mRNA levels are displayed for T-Cell Acute Lymphoblastic Leukemia (T-ALL) compared to Peripheral Blood Mononuclear Cells (PBMCs) obtained from Haferlach et al., [23]. **(B)** *DNMT1*, *DNMT3A* and *DNMT3B* mRNA levels are displayed for non-malignant B-cells (BC) compared to human Burkitt’s lymphoma (BL) obtained from Brune et al., [24]. Boxes indicate the interquartile range; the line within the box represents the median. Whiskers indicate the non-outlier minimum and maximum. Outliers are represented by circles. Significant *p*-values and fold changes are indicated.

To evaluate whether clinical T-ALL and Burkitt’s lymphoma specimens resemble our data from mouse and human tumor cell lines, we took advantage of publically available clinical expression data sets. By analyzing Oncomine data derived from Haferlach et al., [23] we determined that *DNMT1*, *DNMT3A* and *DNMT3B* mRNA expression levels are elevated in clinical T-ALL compared to non-malignant cells (Figure 2A). In T-ALL, *DNMT1* was increased (1.54-fold, *P*=1.78×10^-107^), *DNMT3A* (3.03-fold, *P*=6.35×10^-70^) and *DNMT3B* (1.96-fold, *P*=1.95×10^-34^) compared to Peripheral Blood Mononuclear Cells (PBMCs).

Furthermore, by analyzing Oncomine data derived from Brune et al., [24] we determined that human Burkitt’s lymphoma (BL) specimens significantly overexpress *DNMT1* (1.44-fold, *P*=0.032) and *DNMT3B* (1.46-fold, *P*=4.71×10^-4^) when compared to non-malignant B-cells (BC) obtained from healthy donors (Figure 2B). In contrast, *DNMT3A* mRNA levels were not much higher in Burkitt’s lymphoma (1.09-fold, *P*=0.031) than in normal B-cells, which is consistent with our cell line analysis. Taken together, clinical specimens resembled our data from mouse and human cell line models in regard to *DNMT1* and *DNMT3B* overexpression in T-ALL and Burkitt’s lymphoma.

### Overexpression of DNMT1 and DNMT3B in T-ALL and Burkitt’s lymphoma is MYC-dependent

To determine whether DNMT3B overexpression in T-ALL and Burkitt’s lymphoma cells is MYC-dependent, we took advantage of the tetracycline-regulated *c-MYC* allele in mouse T-ALL (EμSRα-tTA;tet-o-MYC) as well as in a human Burkitt’s lymphoma-like cell line model (P493-6). P493-6 cells are derived from immortalized human peripheral blood B-lymphocytes engineered to harbor a tetracycline-repressible *c-MYC* transgene [25, 26]. P493-6 cells served as a model for MYC activation in Burkitt’s lymphoma. Administering doxycycline (DOX) inactivates MYC transcription in both models in a time- and concentration-dependent manner [6, 27]. Utilizing the T-ALL mouse model, we previously reported that tumors depend on the continuous expression of MYC and undergo tumor regression upon inactivation of the oncogene, thereby exhibiting oncogene addiction [6]. We demonstrated that sustained tumor regression is dependent on cellular senescence, associated with genome-wide changes in chromatin structure and gene expression programs [15, 22, 27–29].

**Figure 3:**
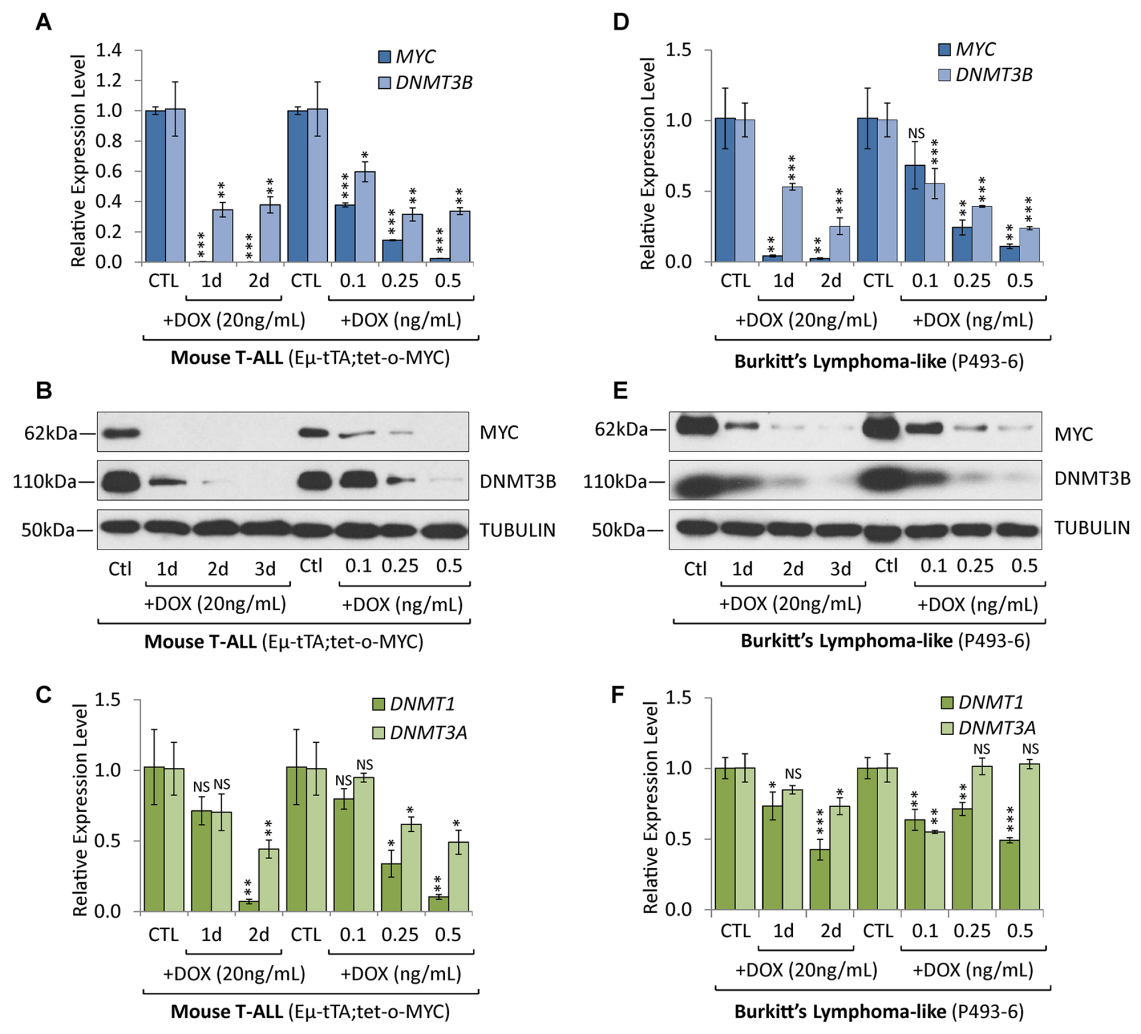
Overexpression of DNMT3B in T-ALL and Burkitt’s lymphoma is MYC-dependent Expression analysis of DNMT3B in T-ALL and Burkitt’s lymphoma models before and upon MYC inactivation. **(A)** RT-qPCR of *MYC* and *DNMT3B*, **(B)** Western blot analysis of MYC and DNMT3B, and **(C)** RT-qPCR of *DNMT1* and *DNMT3A* in T-ALL cells (2833) derived from EμSRα-tTA;tet-o-MYC mice, before and upon inactivation of MYC for 1 and 2 days with 20 ng/mL DOX. Addition of increasing DOX concentrations (0.1-0.5 ng/mL) for 2 days allowed for titration of MYC and DNMT3B levels. **(D)** RT-qPCR of *MYC* and *DNMT3B*, **(E)** Western blot analysis of MYC and DNMT3B, and **(F)** RT-qPCR of *DNMT1* and *DNMT3A* in human Burkitt’s lymphoma-like cells (P493-6) expressing a conditional *c-MYC* allele, before and upon inactivation of MYC for 1 and 2 days with 20 ng/mL DOX. Addition of increasing DOX concentrations (0.1-0.5 ng/mL) for 2 days allowed for titration of MYC and DNMT3B levels. RT-qPCR data was normalized to *UBC* or *RPL13A*; TUBULIN serves as loading control for Western blot. Error bars represent mean ± SEM; *n* = 3; two-tailed Student’s *t*-test: NS = non-significant; *P < 0.05; **P < 0.01; ***P < 0.001.

To determine whether MYC and DNMT expression correlate in a time dependent manner, we treated mouse T-ALL (2833) and human Burkitt’s lymphoma-like cells (P493-6) for 1 and 2 days with 20 ng/mL DOX. In parallel, we titrated MYC expression through increasing DOX concentrations (0.1-0.5ng/mL) for 2 days to measure the concentration-dependence. Subsequent RT-qPCR and Western blot analysis revealed a correlation between MYC and DNMT3B expression levels (Figure 3A–3F). In both T-ALL and Burkitt’s lymphoma-like cells, treatment with 20ng/mL DOX for 1 and 2 days quickly abrogated MYC transcription; DNMT3B showed a decrease of mRNA followed by the corresponding protein. Titrating MYC with 0.1-0.5 ng/mL DOX for 2 days led to a stepwise decrease of *DNMT3B* mRNA and protein levels. *DNMT1* mRNA levels also correlated with the time- and concentration-dependent depletion of MYC in both T-ALL or Burkitt’s lymphoma-like cells; however, while *DNMT3A* mRNA levels followed the same trend in T-ALL, *DNMT3A* levels were less consistent with the titration of MYC in Burkitt’s lymphoma-like cells. Taken together, DNMT1 and DNMT3B expression exhibited a correlation to MYC levels in both mouse T-ALL and human Burkitt’s lymphoma models.

### *DNMT1* and *DNMT3B* are direct transcriptional targets of MYC in T-ALL and Burkitt’s lymphoma

To determine whether *DNMT1* and *DNMT3B* transcription in T-ALL and Burkitt’s lymphoma-like cells is directly regulated by oncogenic MYC, we performed chromatin immunoprecipitation (ChIP) analysis measuring MYC binding to the genomic loci of *DNMT1*, *DNMT3A* and *DNMT3B*.

**Figure 4:**
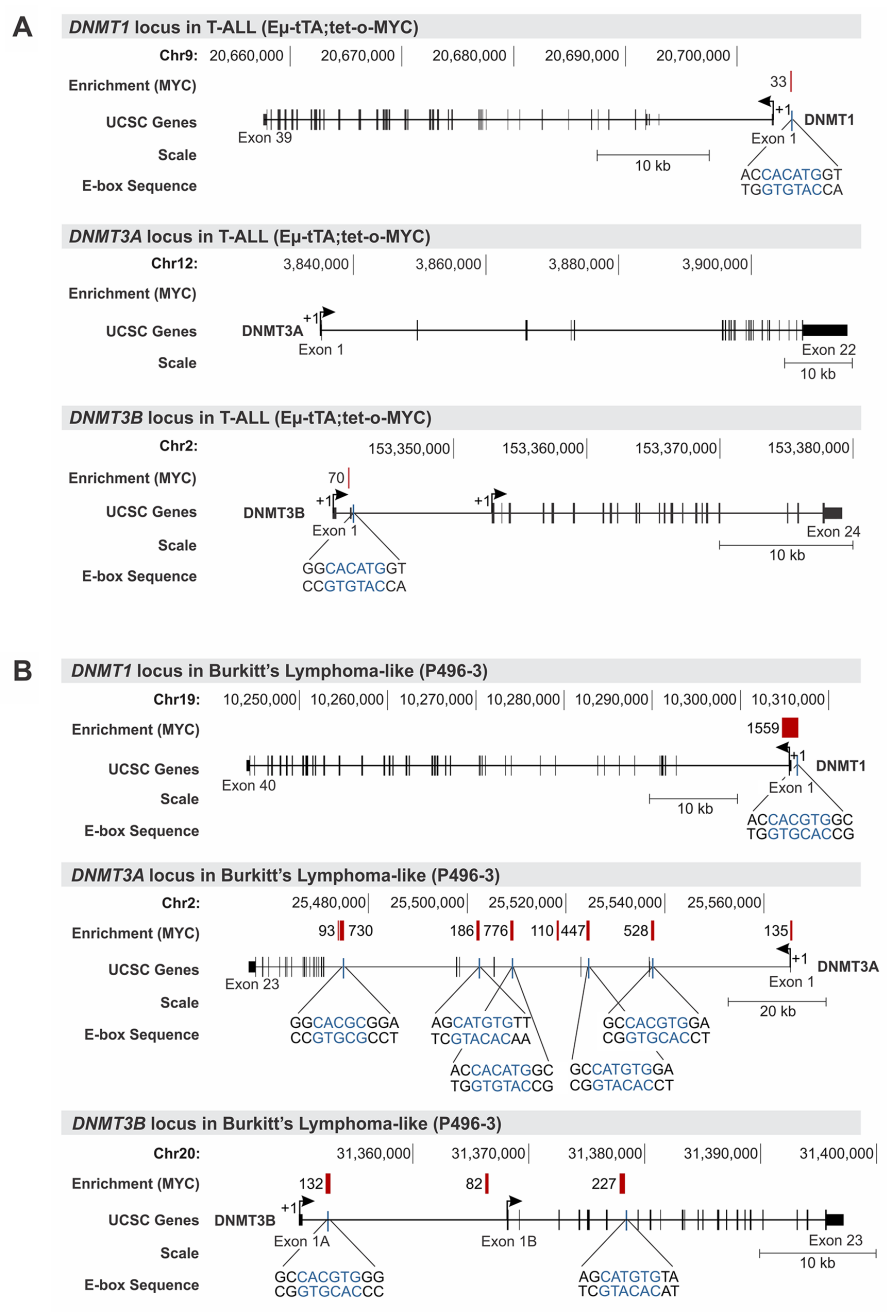
MYC occupies the promoter of *DNMT1* and *DNMT3B* in T-ALL and Burkitt’s lymphoma Chromatin immunoprecipitation (ChIP) analysis indicates high relative enrichment for MYC at the *DNMT1* and *DNMT3B* promoters in mouse T-ALL and human Burkitt’s lymphoma-like cells. **(A)** Agilent mouse promoter microarrays covering -5.5kb to +2.5kb of the transcription start site were used for ChIP-chip analysis. MYC ChIP-chip data for mouse T-ALL cells (EμSRα-tTA;tet-o-MYC) indicating enrichment for the DNMT1, DNMT3A and DNMT3B locus. **(B)** MYC ChIP-seq data for Burkitt’s lymphoma-like cells (P493-6) obtained from Sabo et al. [30] indicating enrichment scores for *DNMT1*, *DNMT3A* and *DNMT3B*. Traces for *DNMT1*, *DNMT3A* and *DNMT3B* were generated based on reference genome mm8 and hg19, respectively, using the UCSC Genome Browser. The chromosomal location is indicated in bp, and the scale in kb. MYC binding peaks are displayed as red vertical bars; numbers represent the relative fold enrichment for MYC. E-box sequences and their location are shown in blue for the vicinity of MYC binding peaks. Exons are displayed as black vertical bars, the UTR is represented by a black line, and the transcription start site (TSS) is marked by an arrow indicating the direction of transcription.

For MYC-driven T-ALL cells (2833) derived from EμSRα-tTA;tet-o-MYC transgenic mice we utilized ChIP followed by promoter microarray hybridization (ChIP-chip) (Figure 4A). We found MYC to be significantly enriched at the *DNMT1* locus directly upstream of exon 1 (relative enrichment: 33) and at the *DNMT3B* locus downstream of exon 1 (relative enrichment: 70). A high resolution view of the *DNMT1*, *DNMT3A* and *DNMT3B* loci in T-ALL including the location of the ChIP microarray probes is shown in Supplementary Figure 2. We validated the MYC binding peaks identified by ChIP-chip using direct ChIP-qPCR analysis for *DNMT1* and *DNMT3B* in T-ALL (Supplementary Figure 3). To determine whether Burkitt’s lymphoma mimic T-ALL in this regard, we took advantage of publically available ChIP-seq data sets for high MYC-expressing P493-6 cells from Sabo et al. [30] (Figure 4B). Consistent with the results in T-ALL, we found MYC to occupy the *DNMT1* promoter in P493-6 cells upstream of exon 1 (relative enrichment: 1,559), and *DNMT3B* downstream of exon 1 (relative enrichment: 132) as well as at two more sites in the gene body, which was not covered by the promoter array analysis in mouse T-ALL. While the *DNMT3A* locus did not show any significant enrichment for MYC in T-ALL, we found multiple sites in the gene body to be bound by MYC in P493-6 cells.

**Figure 5:**
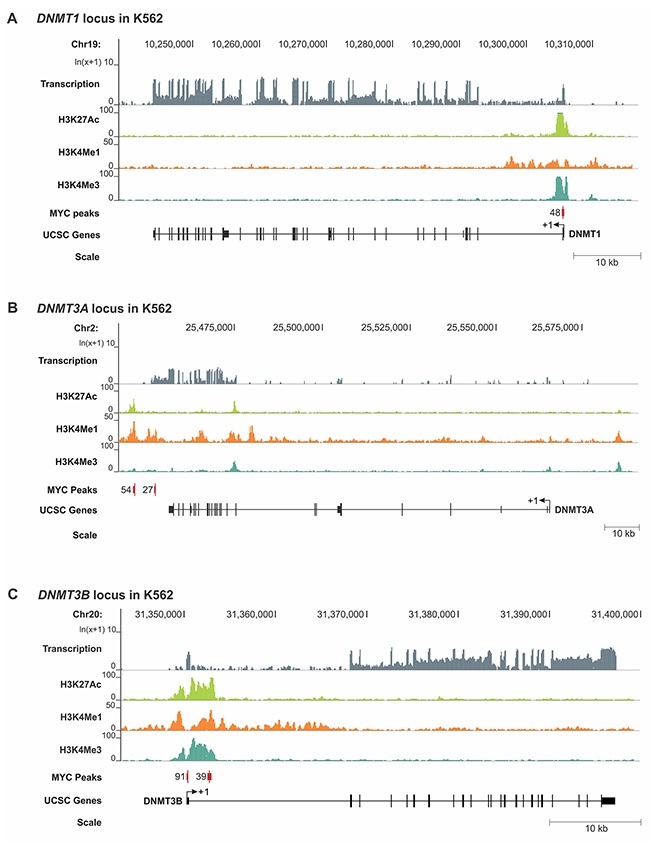
RNA-seq and ChIP-seq analysis for H3K27Ac, H3K4Me1, H3K4Me3 and MYC for the *DNMT1*, *DNMT3A* and *DNMT3B* loci in CML cells Publically available RNA-seq (transcription) and ChIP-seq data (IP: H3K27Ac, H3K4Me1, H3K4Me3 and MYC) for human CML cells (K562) were analyzed to display the relative enrichment (Y axis) for the genomic loci (X axis) of **(A)** *DNMT1* **(B)** *DNMT3A* and **(C)** *DNMT3B*. ENCODE [32] data sets: RNA-seq (GSM958729), H3K27Ac (GSM733656), H3K4Me1 (GSM733692), H3K4Me3 (GSM733680), MYC (GSM935516). Enrichment peaks for MYC are displayed as red vertical bars; the number represents the signal value. The chromosomal location is indicated in bp, and the scale in kb. Exons are displayed as black vertical bars, the UTR is represented by a line, and the transcription start site (TSS) is marked by an arrow indicating the direction of transcription. Schematic was generated based on reference genome hg19 using the UCSC Genome Browser.

To further elucidate the mechanism of transcriptional *DNMT1* and *DNMT3B* regulation by MYC, we retrieved the genomic DNA sequences of the MYC binding peaks including surrounding sequences from the UCSC genome browser and performed an E-box motif search using the transcription factor binding profile software, JASPAR [31]. We identified canonical E-box sequences in close proximity to the MYC enrichment peak near the transcription start site (TSS) of *DNMT1* and *DNMT3B* in both T-ALL, and Burkitt’s lymphoma-like cells (Figure 4A and 4B). To gain further insights into whether MYC binding sites are located in a regulatory region, we extended our analysis to include the chromatin status of *DNMT1*, *DNMT3A* and *DNMT3B* overlayed with MYC binding peaks. For that purpose, we turned to publically available ENCODE [32] Chip-seq and RNA-seq data sets for human Chronic Myeloid Leukemia (CML) (K562) and human embryonic stem cells (H1-hESCs) displaying the status of H3K27Ac, H3K4Me1, H3K4Me3 and MYC for the genomic loci of *DNMT1*, *DNMT3A* and *DNMT3B* (Figure 5 and Supplementary Figure 4). We found that in CML the MYC binding site upstream of *DNMT1* exon 1 is enriched for the active chromatin marks H3K27Ac and H3K4Me3, as well as for H3K4Me1, which is associated with enhancer elements [33, 34]. Similarly, the MYC binding site downstream of *DNMT3B* exon 3 is enriched for all three marks, H3K27Ac, H3K4Me1 and H3K4Me3. In contrast, *DNMT3A* lacked MYC binding peaks and also did not indicate enrichment for H3K27Ac, H3K4Me1 or H3K4Me3 near the TSS.

In summary, the genomic location analysis together with expression profiling indicates that MYC directly binds to *DNMT1* and *DNMT3B* in T-ALL and Burkitt’s lymphoma, thereby establishing both *DNMTs* as direct transcriptional targets of the MYC oncoprotein. Furthermore, the distribution of H3K27Ac, H3K4Me1 or H3K4Me3 suggests that the MYC binding sites for *DNMT1* and *DNMT3B* are located within active promoter/enhancer regions further strengthening the model of a direct transcriptional regulation.

### Suppression of endogenous MYC in human T-ALL and Burkitt’s lymphoma cell lines leads to decreased DNMT3B expression

**Figure 6:**
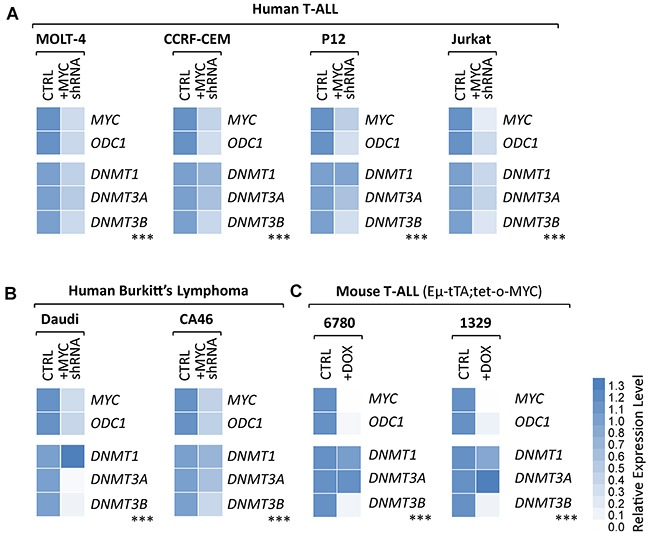
Knock-down of endogenous *MYC* leads to diminished *DNMT3B* expression levels in human T-ALL and Burkitt’s lymphoma cell lines shRNA-mediated knock-down of endogenous *MYC* in human T-ALL and Burkitt’s lymphoma cell lines. RT-qPCR analysis of *MYC* and its canonical target gene, *ODC1*, serving as control, as well as *DNMT1*, *DNMT3A*, *DNMT3B*. **(A)** Human T-ALL (MOLT-4, CCRF-CEM, P12-Ichikawa and Jurkat), **(B)** human Burkitt’s lymphoma (Daudi and CA46) cell lines before and upon tetracycline-inducible knock-down of MYC (+MYC shRNA) for 2 days. **(C)** Mouse T-ALL (EμSRα-tTA;tet-o-MYC) cell lines 6780 and 1329 before and upon inactivation of MYC (+DOX). RT-qPCR data was normalized to UBC. *n* = 3; two-tailed Student’s *t*-test: ***P < 0.001.

To determine whether DNMT3B overexpression levels in human B- and T-cell lymphoma cell lines depend on high levels of endogenous MYC, we analyzed a panel of human T-cell lymphoma and Burkitt’s lymphoma cell lines before and upon knock-down of *MYC* via tetracycline-inducible shRNA (Figure 6A, 6B and Supplementary Figure 5). Jurkat, MOLT-4, P12-Ichikawa and CCRF-CEM (T-ALL), and Daudi and CA46 (Burkitt’s lymphoma) were infected with either a shRNA targeting human *MYC* (+MYC shRNA)or a scrambled control shRNA (CTRL). Before and upon induction of the shRNA through addition of 100 ng/mL DOX to the culture medium for 2 days, the expression level of *MYC*, its canonical target gene *ODC1*, as well as *DNMT1*, *DNMT3A* and *DNMT3B* were determined by RT-qPCR. In parallel, we performed the same analysis on two additional T-ALL cell lines (6780 and 1329) derived from EμSRα-tTA;tet-o-MYC mice, before and upon suppression of transgenic *MYC* for 1 day via 20 ng/mL DOX (Figure 6C). *MYC* and its canonical target gene *ODC1* were drastically decreased confirming efficient MYC knock-down in all cell lines. All analyzed cell lines show a consistent significant decrease in *DNMT3B* mRNA level upon MYC inactivation. Taken together, the expression analysis indicates a direct correlation between MYC and *DNMT3B* expression levels in human B- and T-cell lymphoma. In combination with the promoter binding assay, the expression analysis provides evidence for a direct transcriptional of regulation *DNMT3B* by MYC in human T-ALL and Burkitt’s lymphoma.

### Loss of DNMT3B function decreases tumor cell proliferation

**Figure 7:**
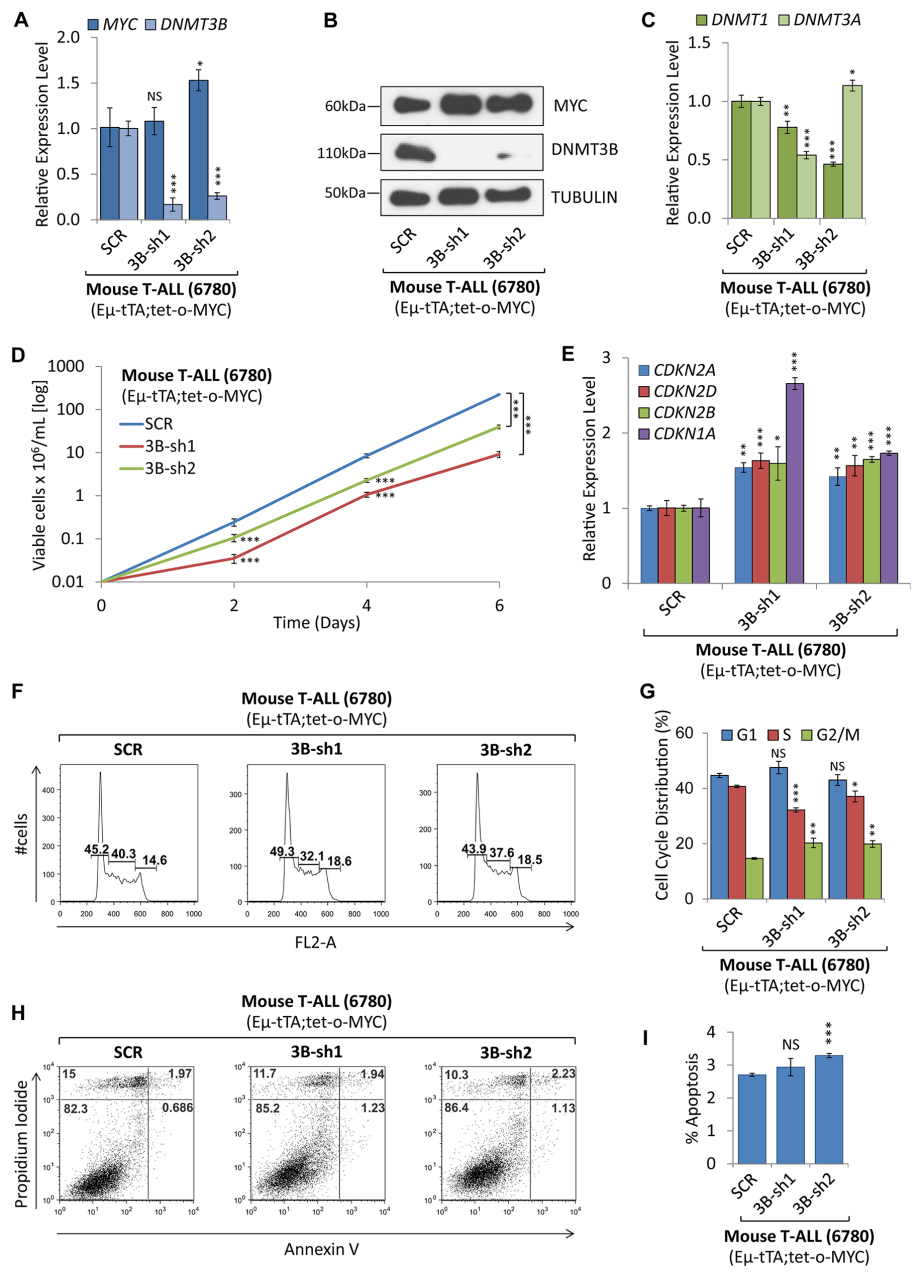
shRNA-mediated knock-down of *DNMT3B* decreased tumor cell proliferation shRNA-mediated knock-down of *DNMT3B* in mouse T-ALL (EμSRα-tTA;tet-o-MYC). T-ALL cells (6780) upon shRNA-mediated knock-down of *DNMT3B* using two distinct sequences (3B-sh1 and 3B-sh2), were compared to control cells (SCR). **(A)** RT-qPCR analysis of *MYC* and *DNMT3B*. **(B)** Western blot analysis of MYC and DNMT3B; TUBULIN serves as loading control. **(C)** RT-qPCR analysis of *DNMT1* and *DNMT3A*. **(D)** Growth curve comparing viable cell counts. **(E)** RT-qPCR analysis of cell cycle-dependent kinase inhibitors *CDKN2A, CDKN2D, CDKN2B, and CDKN1A*. **(F)** Flow cytometric cell cycle analysis using propidium iodide (PI) staining. **(G)** Cell cycle distribution (G1, S and G2/M) displayed in percent. **(H)** Flow cytometric analysis of apoptosis using Annexin V/PI staining. Flow cytometry profile of Annexin V staining (X axis) and PI (Y axis) is shown for representative samples. The lower right quadrant indicates the percentage of early apoptotic cells in each condition; the upper right quadrant indicates the percentage of late apoptotic cells. **(I)** Apoptotic cells (Annexin V-positive cells) are displayed as the percentage of gated cells. Error bars represent mean ± SEM; *n* = 3; two-tailed Student’s *t*-test: *P < 0.05; **P < 0.01; ***P < 0.001.

To determine whether loss of DNMT3B function affects tumor cell proliferation and viability we carried out shRNA-mediated knock-down as well as pharmacologic inhibition of DNMT3B in MYC-driven T-ALL cells. We compared T-ALL cells upon stable shRNA-mediated knock-down of *DNMT3B* to scrambled shRNA control. RT-qPCR and Western blot analysis confirmed a decrease of DNMT3B upon knock-down using two different shRNA sequences, 3B-sh1 -5.95-fold (*P*<0.001) and 3B-sh2 -3.82-fold (*P*<0.001). MYC expression did not change significantly in 3B-sh1 but increased 1.5-fold in 3B-sh2 (*P*<0.05) (Figure 7A and 7B). *DNMT1* and *DNMT3A* mRNA levels decreased -1.28-fold (*P*<0.01) and -2.16-fold (*P*<0.001) for 3B-sh1, respectively, compared to the control cells (Figure 7C). In parallel, we analyzed cell growth, viability and cell cycle. The growth curve indicates that loss of DNMT3B function decreases tumor cell proliferation significantly (Figure 7D). RT-qPCR analysis revealed a significant upregulation of the direct DNMT3B target, *CDKN1A* (p21CIP1), in 3B-sh1 and 3B-sh2 cells (2.66-fold, *P*<0.001 and 1.73-fold, *P*<0.001, respectively) [35]. Furthermore, we found a number of cycline-dependent kinase inhibitors to increase significantly upon knock-down of DNMT3B. *CDKN2B* (p15INK4b) increased 1.60- and 1.65-fold (*P*<0.05 and *P*<0.001), *CDKN2A* (p16INK4a) increased 1.42- and 1.54-fold (*P*<0.01 and *P*<0.01), and *CDKN2D* (p19INK4d) increased 1.63- and 1.57-fold (*P*<0.001 and *P*<0.01). Flow cytometric cell viability and cell cycle analysis based on propidium iodide (PI) confirmed a decrease in cell proliferation, indicating a decrease in S phase cells from 40.3% for SCR to 32.1% (*P*<0.001) for 3B-sh1 and 37.6% for 3B-sh2 (*P*<0.05) (Figure 7F and 7G). In parallel, we measured apoptosis by Annexin V and PI staining followed by flow cytometric analysis as shown in Figure 7H and 7I. While we did not observe a statistically significant change for 3B-sh2, apoptosis in 3B-sh1 cells increased by 1.9% (*P*<0.01). In summary, we conclude that loss of DNMT3B expression in T-ALL leads to increase of cycline-dependent kinase inhibitors and diminished cell proliferation through cell cycle arrest, rather than apoptosis.

**Figure 8:**
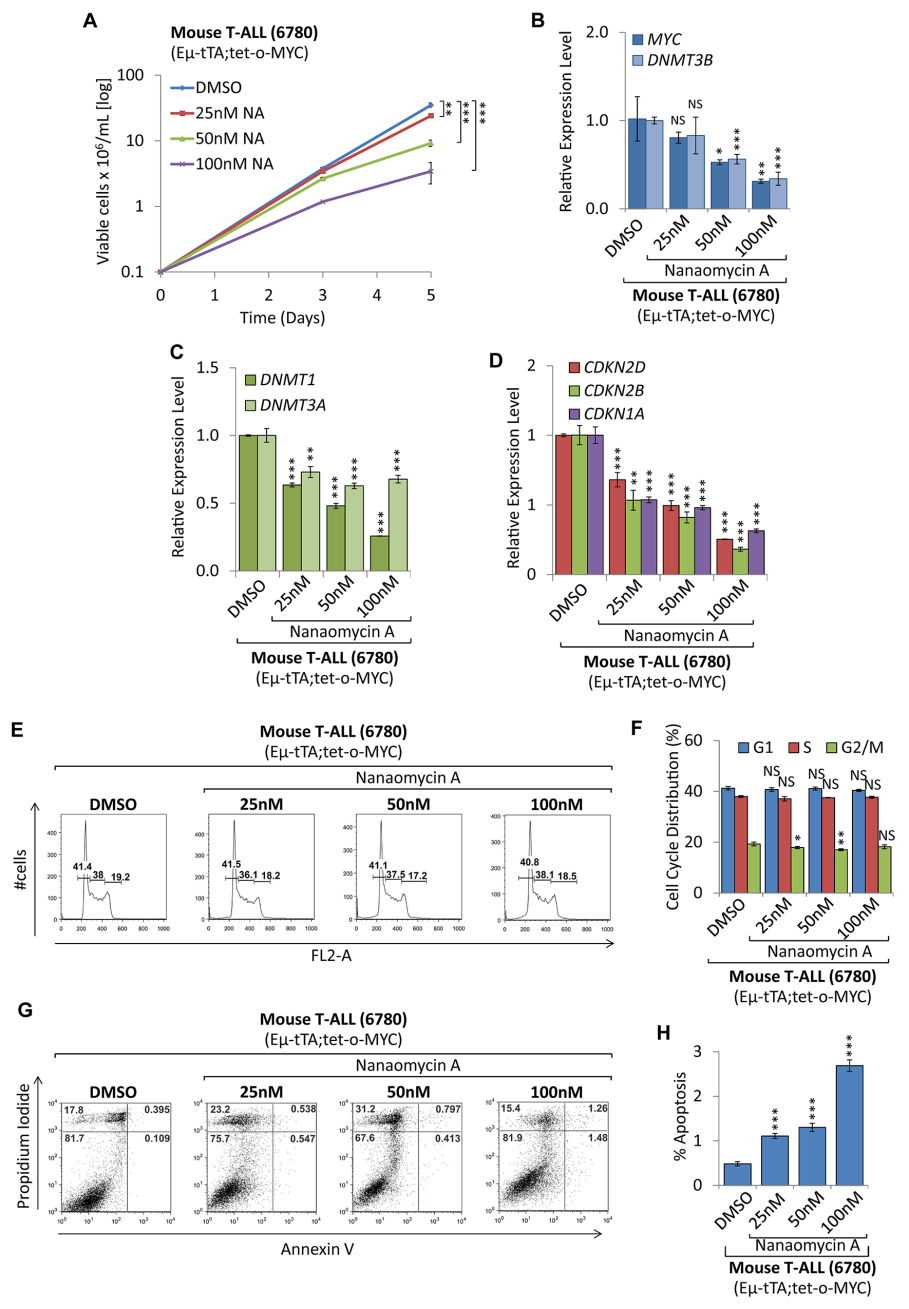
Pharmacologic inhibition of DNMT3B function decreased tumor cell proliferation Pharmacologic inhibition of DNMT3B in mouse T-ALL (EμSRα-tTA;tet-o-MYC). T-ALL (6780) were treated with 25nM, 50nM and 100nM of the DNMT3B inhibitor Nanaomycin A, compared to DMSO control. **(A)** Growth curve comparing viable cell counts are displayed on a logarithmic scale. **(B)** RT-qPCR analysis of MYC and DNMT3B. **(C)** RT-qPCR analysis of DNMT1 and DNMT3A. **(D)** RT-qPCR analysis of cell cycle-dependent kinase inhibitors *CDKN2D, CDKN2B, and CDKN1A*. RT-qPCR was normalized to *UBC*. **(E)** Flow cytometric cell cycle analysis based on propidium iodide (PI) staining. **(F)** Cell cycle distribution (G1, S and G2/M) displayed in percent. **(G)** Flow cytometric analysis of apoptosis based on Annexin V/PI staining. Flow cytometry profile of Annexin V staining (X axis) and PI (Y axis) is shown for representative samples. The lower right quadrant indicates the percentage of early apoptotic cells in each condition; the upper right quadrant indicates the percentage of late apoptotic cells. **(H)** Apoptotic cells (Annexin V-positive cells) are displayed as the percentage of gated cells. Error bars represent mean ± SEM; *n* = 3; two-tailed Student’s *t*-test: NS = non-significant; *P < 0.05; **P < 0.01; ***P < 0.001.

To compare shRNA mediated knock-down of DNMT3B to pharmacologic inhibition, we treated mouse T-ALL cells with the DNMT3B inhibitor Nanaomycin A [36]. Treatment with 25, 50, and 100nM Nanaomycin A (NA) diminished cell proliferation and viability drastically compared to DMSO treated control cells (Figure 8A), indicating a dose-dependent effect on tumor cells. This was accompanied by a decrease of *MYC*, *DNMT3B*, *DNMT3A*, and *DNMT1* expression (Figure 8B and 8C). In contrast to the shRNA-mediated knock-down of DNMT3B, NA caused a significant downregulation of *CDKN1A*, (-1.86, -2.08, -3.18-fold, respectively (*P*<0.001)), *CDKN2D* (-1.47, -2.02, -3.95-fold respectively (*P*<0.001)) and *CDKN2B* mRNA levels (-1.88, -2.44, -5.50-fold, respectively, (*P*<0.01 and *P*<0.001)) (Figure 8D). Flow cytometric cell viability and cell cycle analysis based on PI staining did not show a significant decrease in cell proliferation (Figure 8E and 8F). However, Annexin V/PI staining revealed a dose-dependent increase in apoptotic cells from 0.48% to 2.69% (*P*<0.001) upon 100nM NA treatment for 2 days (Figure 8G and 8H), suggesting cell death rather than cell cycle arrest as the mechanism of action. Contrasting the shRNA-mediated knock-down result, we conclude that the latter outcome has to be contributed to a broad cytotoxic effect. Treatment of non-malignant human embryonic kidney (HEK293T) cells, which don’t express detectable levels of DNMT3B, with NA led to a significant, but less drastic reduction in cell proliferation (25nM NA reduced T-ALL proliferation by 31.1%, while affecting HEK293T cells by 17.5%) (Supplementary Figure 6). While the only commercially available DNMT3B inhibitor (NA) exhibited a broad cytotoxic response, we conclude that specific shRNA-mediated knock-down of DNMT3B decreased tumor cell proliferation by upregulating tumor suppressor genes and triggering a cell cycle arrest rather than cell death. Taken together, this indicates that DNMT3B function is required for MYC-driven tumor maintenance in T-ALL.

### DNMT3B knock-down affects genome-wide DNA methylation in T-ALL

**Figure 9:**
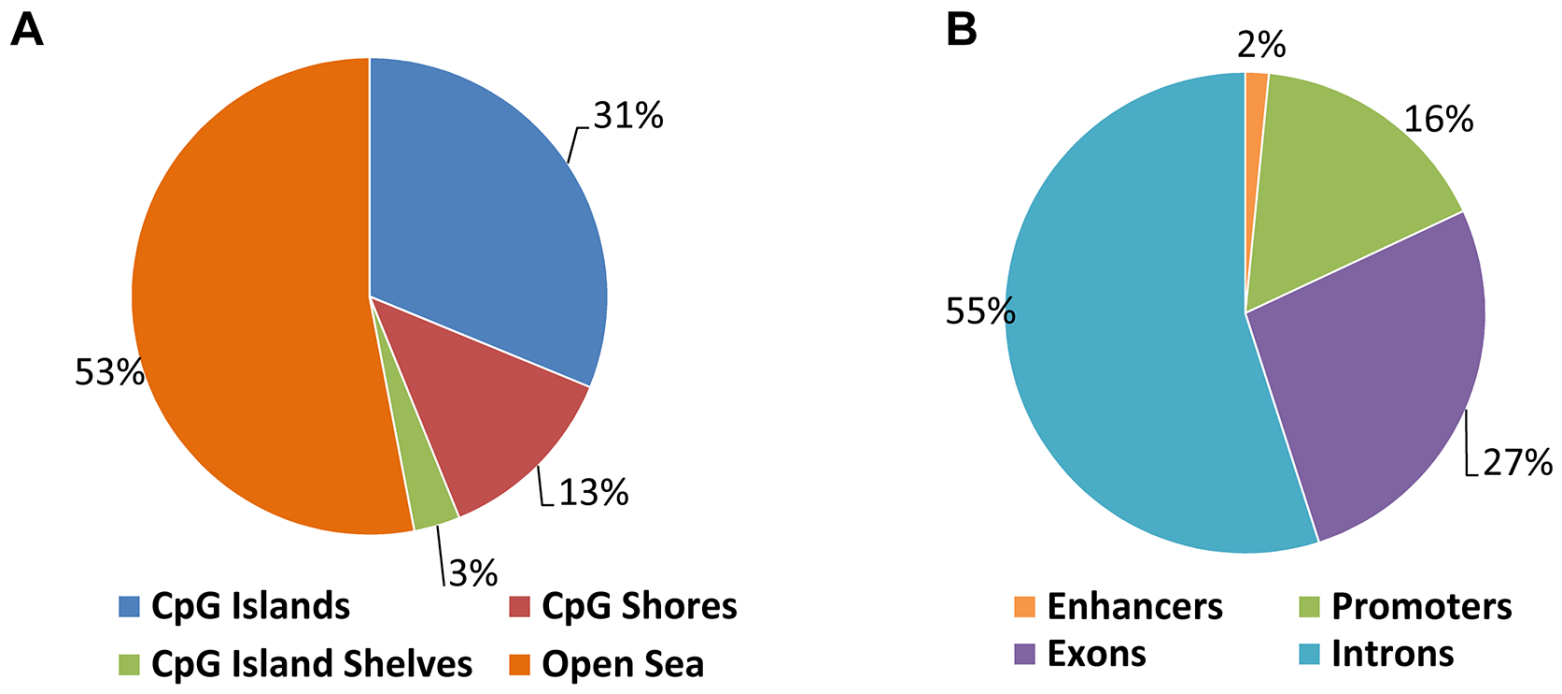
Genomic distribution of differentially methylated CpGs in T-ALL before and upon DNMT3B knock-down T-ALL (EμSRα-tTA;tet-o-MYC) cells were analyzed upon shRNA-mediated knock-down of DNMT3B (6780 3B-sh2) compared to scrambled control cells (6780 SCR) by reduced representation bisulfite sequencing (RRBS). **(A)** Genomic distribution of differentially methylated CpGs indicating CpG islands, CpG island shelves, CpG island shores and open sea. **(B)** Genomic distribution of differentially methylated CpGs indicating enhancers, exons, intergenic regions, introns and promoters.

**Figure 10:**
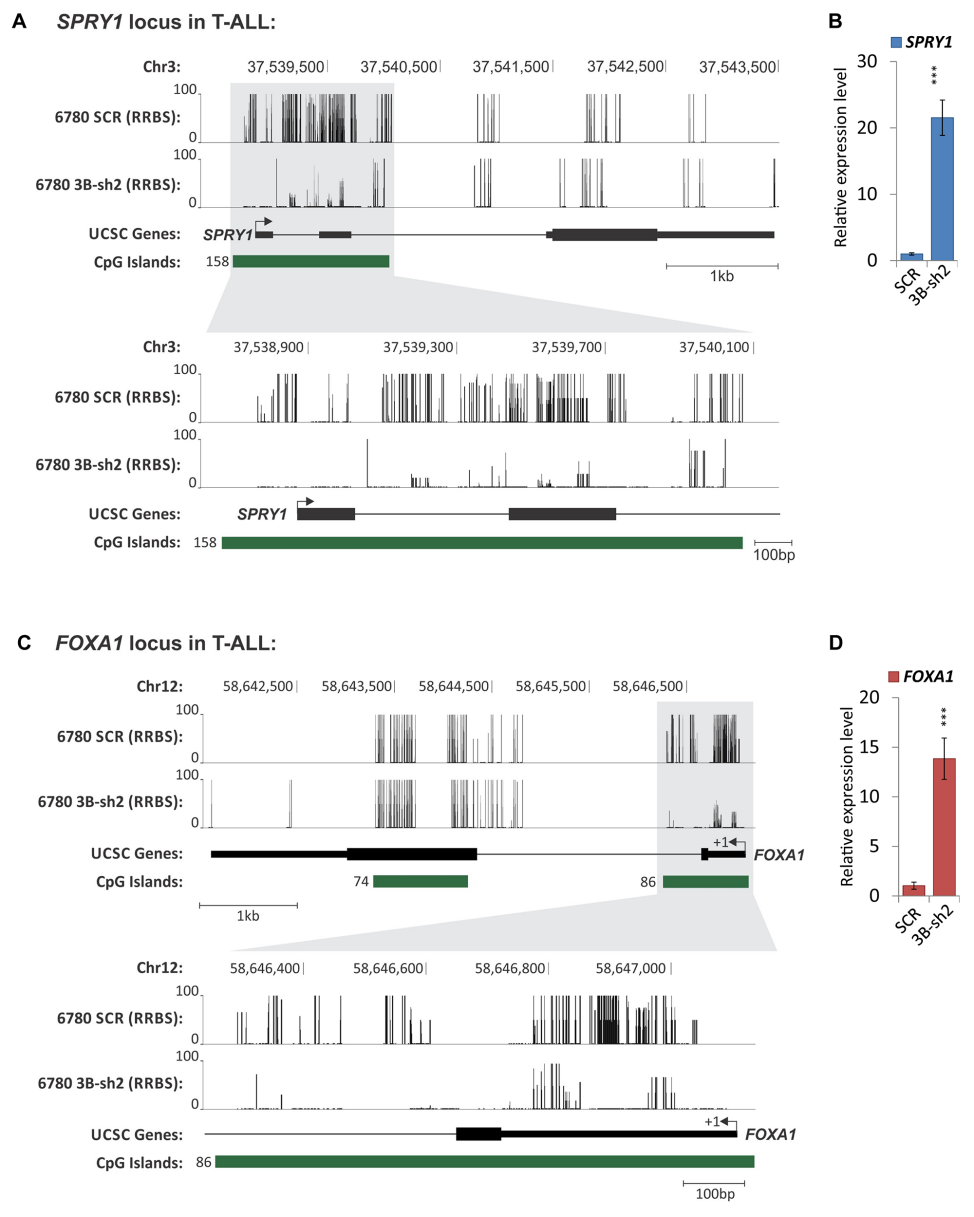
Gene-specific DNA methylation analysis of T-ALL before and upon DNMT3B knock-down using RRBS T-ALL (EμSRα-tTA;tet-o-MYC) cells were analyzed upon shRNA-mediated knock-down of DNMT3B (6780 3B-sh2) compared to scrambled control cells (6780 SCR) by RRBS. DNA methylation is displayed for the genomic loci of **(A)** *SPRY1* (*sprouty RTK signaling antagonist 1*), **(C)** *FOXA1* (*forkhead box A1*). Methylation level of CpG nucleotides is indicated on the Y axis; genomic location is indicated on the X axis. The chromosomal location is indicated in bp, and the scale in kb. Exons are displayed as black vertical bars, the UTR is represented by a line, and the transcription start site (TSS) is marked by an arrow indicating the direction of transcription. CpG islands are displayed as green bars; the number of CpGs per island is indicated. Schematic was generated based on reference genome mm9 using the UCSC Genome Browser. RT-qPCR expression profiling of **(B)** *SPRY1*, and **(D)** *FOXA1* before (SCR) and upon knock-down of DNMT3B (3B-sh2) in T-ALL (6780) cells. RT-qPCR was normalized to *UBC*. Error bars represent mean ± SEM; *n* = 3; two-tailed Student’s *t*-test: ***P < 0.001.

To determine the effect of loss of DNMT3B function on genome-wide DNA methylation we applied reduced representation bisulfite sequencing (RRBS). We compared the above described MYC-driven T-ALL cells (EμSRα-tTA;tet-o-MYC) stably expressing a *DNMT3B*-specific shRNA (6780 3B-sh2) to cells harboring a scrambled shRNA (6780 SCR) as control (Figures 9 and 10 and Supplementary Figure 7). We generated 35–40 million Illumina sequencing reads for each sample. Of these, 70% were successfully mapped to either strand of the mouse genome (mm9). We were able to consistently determine the methylation status of approximately 1.9–2.1 million CpGs. Over 20,000 CpG islands (CGIs), which accounts for more than 80% of annotated CGIs in the genome, were examined. The genomic distribution of differentially methylated CpGs between 6780 SCR and 3B-sh2 indicates genome-wide changes including CpG islands, CpG island shelves, CpG island shores and open sea (Figure 9A), as well as enhancers, exons, intergenic regions, introns and promoters (Figure 9B). To identify differentially methylated regions (DMRs) in this data set, we performed a genome-wide, unbiased DMR detection using a complete tiling of the mouse genome in 1,000 bp windows with a percent methylation difference cutoff of 25% and q-value of 0.01. We found a total of 30,330 DMRs that were hyper- or hypomethylated upon DNMT3B knock-down. Thus, the genome-wide analysis indicates global changes in the DNA methylation pattern of T-ALL cells upon loss of DNMT3B.

Within the RRBS data set, we identified 6,224 genes that were differentially methylated within their promoters and 8,552 genes differentially methylated within their exonic regions upon knock-down of DNMT3B. Furthermore, we found 8,645 defined CpG islands which were differentially methylated. To show the effect of DNMT3B knock-down on the DNA methylation of specific genes, we display the distribution of methylated CpGs in 6780 SCR vs. 3B-sh2 across four candidates: *SPRY1* (*sprouty RTK signaling antagonist 1*) and *FOXA1* (*forkhead box A1*) (Figure 10A and 10C), *SOX12* (*SRY (sex determining region Y)-box 12*) and *GLIS2* (*GLIS family zinc finger 2*) (Supplementary Figure 7A and 7C). For all shown genes, loss of DNMT3B expression was associated with a marked decrease of DNA methylation in CpG islands located near the transcription start site (TSS). We conclude that loss of DNMT3B can reverse promoter/CpG island hypermethylation in T-ALL. To show the functional consequences of hypomethylation events in regulatory regions, we performed expression profiling for the above genes. Indeed, RT-qPCR expression analysis indicates a marked increase in mRNA for *SPRY1* (21.53-fold, *P*<0.001) and *FOXA1* (13.85-fold, *P*<0.001) (Figure 10B and 10D), *SOX12* (15.29-fold, *P*<0.001) and *GLIS2* (4.19-fold, *P*<0.001) (Supplementary Figure 7B and 7D) upon loss of DNMT3B.

In summary, we conclude that DNMT3B contributes to tumor maintenance of MYC-driven T-ALL cells through its effects on DNA methylation, and that loss of DNMT3B causes the reactivation of gene transcription through reversing promoter/CpG island methylation.

## DISCUSSION

Aberrant DNA methylation is an important feature of tumor cells. However, how tumor cell-specific DNA methylation patterns are established and maintained through the coordinated action of DNA methylating enzymes remains less understood. Here, we report that in T-ALL and Burkitt’s lymphoma MYC directly caused the overexpression of both DNMT1 and DNMT3B. This suggests for the first time that the MYC oncoprotein controls DNA methylation patterns in a genome-wide fashion via DNMTs.

To investigate the role of DNMTs in MYC-driven tumor maintenance, we performed expression profiling on T-ALL and Burkitt’s lymphoma compared to non-malignant tissue. We found that DNMT1 and DNMT3B to be consistently overexpressed in transgenic models, human tumor cell lines, as well as clinical specimens. In contrast, DNMT3A expression was not consistent in both MYC-driven tumor types. Burkitt’s lymphoma did not display increased DNMT3A levels, implicating a differential mechanism of regulation. Our results are consistent with reports that DNMT activity is often elevated in tumor cells and is subsequently implicated in disease progression. All three DNMT enzymes have been reported to be overexpressed in hematological malignancies such as acute myelogenous leukemia (AML), chronic myelogenous leukemia (CML), diffuse large B-cell lymphoma (DLBCL) as well as mantel cell lymphoma (MCL) [37–40]. In AML, *microRNA-29b* targets *DNMT3A* and *DNMT3B* directly and *DNMT1* indirectly thereby inducing global DNA hypomethylation and tumor suppressor gene expression [41]. Interestingly, increased DNMT3B levels are a negative prognostic factor in AML and DLBCL [42–45]. It remains to be seen whether DNMT1 or DNMT3B overexpression is also associated with poor clinical outcome in T-ALL and Burkitt’s lymphoma.

Further examination of the underlying regulatory mechanism in T-ALL and Burkitt’s lymphoma revealed a direct correlation of DNMT1 and DNMT3B expression with high MYC levels, which led us to assess whether transcriptional regulation is MYC-dependent. Indeed, we found that DNMT1 and DNMT3B are overexpressed in a MYC-dependent manner. The tetracycline-regulated *MYC* transgene in a mouse T-ALL (EμSRα-tTA;tet-o-MYC) and human Burkitt’s lymphoma (P493-6) model, allowed the time- and concentration-dependent inactivation of MYC, causing both DNMT1 and DNMT3B expression to decrease step-wise exhibiting a direct correlation with MYC levels. Subsequent ChIP analysis revealed that MYC occupies sites within the *DNMT1* and *DNMT3B* regulatory regions, suggesting a direct transcriptional regulation in mouse T-ALL and human Burkitt’s lymphoma-like cells. Sequence analysis identified canonical E-box sites for the MYC enrichment peaks for both *DNMT1* and *DNMT3B* loci, supporting the model of direct MYC binding. Extending our analysis to include the chromatin status of *DNMT1*, and *DNMT3B*, we found that MYC binding peaks are enriched for H3K27Ac and H3K4Me3 typically placed near active regulatory promoter elements. Furthermore, we found H3K4Me1 which linked to distal regulatory regions to co-localize, overall strengthening our model that MYC directly upregulates *DNMT1* and *DNMT3B* transcription. Knock-down of endogenous MYC in human T-ALL and Burkitt’s lymphoma cell lines supports above findings, indicating that DNMT3B overexpression directly depends on high levels of MYC. Only few direct transcriptional regulators of *DNMT3B* such as SP1, SP3 or FOXO3A [46, 47] have been identified and only few that are known have such a clear and important link to cancer (for example, FOXO3A negatively regulates *DNMT3B* promoter activity in lung cancer [47]). Hence, our finding that MYC directly increases transcription of *DNMT3B* in T-ALL and Burkitt’s lymphoma reveals a novel mechanism of deregulation in cancer. The notion that the effect of MYC on DNMT1 expression is less consistent than on DNMT3B might indicate that DNMT1 levels are the reflection of cell proliferation rather than MYC activity directly. Considering the role of DNMT1 as maintenance DNMT this fits the working model. However, since in T-ALL MYC levels directly affect proliferation, the two are difficult to dissect. In combination with the promoter binding assay, the expression analysis provides evidence that increased DNMT3B expression is directly dependent on a positive transcriptional regulation by MYC.

Above findings suggested that a complex interplay of DNMTs is required for MYC-driven tumor maintenance. Indeed, shRNA-mediated loss of DNMT3B expression impaired cell proliferation in T-ALL. While this was associated with a significant decrease of S phase cells, we did not see a drastic increase in apoptotic cells. Supporting the notion of a cell cycle arrest rather than cell death as mechanism, we found a number of tumor suppressor genes, including *CDKN2B* (p15INK4b), *CDKN2A* (p16INK4a), *CDKN2D* (p19INK4d), and *CDKN1A* (p21CIP1) to increase upon DNMT3B knock-down. This indicates that the above genes are either directly or indirectly suppressed by DNMT3B during T-ALL maintenance, suggesting that DNMT3B acts as a tumor promoter in this context. Controversially, recent reports indicate that DNMT3B functions as tumor suppressor during tumor initiation. Knockout of *DNMT3B* accelerates the initiation of MYC-driven T- and B-cell lymphoma in mouse models (EμSRα-tTA;tet-o-MYC and Eμ-MYC) [48–50], while increased DNA methylation of DNMT3B targets delays leukemogenesis [51]. Similarly, methylation-independent repression of DNMT3B contributes to oncogenic activity of DNMT3A in MYC-induced T-cell lymphomagenesis [52]. Conversely, loss of *DNMT1* has been shown to delay the onset of T-cell lymphoma by suppressing tumor cell proliferation [53]. The divergence to our results might be explained by the circumstance that above studies are based on knockout mice that develop MYC-driven tumors in the absence of DNMT3B. This suggests a differential role of DNMT3B during tumor initiation versus maintenance. However, further studies are required to answer whether individual DNMTs can indeed function as tumor promoter or suppressor depending on the context.

Gaining mechanistic insight into the role of DNMT3B in establishing and maintaining DNA methylation patterns in T-ALL cells, we found that loss of DNMT3B expression caused genome-wide changes associated with changes in gene expression. The approximately 30,000 differentially methylated regions that were hyper- or hypomethylated are distributed across CpG islands, CpG island shelves, CpG island shores and open sea, as well as gene regulatory elements like enhancers, exons, intergenic regions, introns and promoters. More than 6,200 genes were differentially methylated within their promoters and more than 8,600 CpG islands were differentially methylated. For four candidates, *SPRY1*, *FOXA1*, *SOX12*, and *GLIS2*, we show that loss of DNMT3B expression was associated with a marked decrease of DNA methylation in CpG islands that are located near the transcription start site, accompanied by reactivation of the corresponding genes. Hence, we conclude that DNMT3B contributes to tumor maintenance of MYC-driven T-ALL through its effects on DNA methylation, and that loss of DNMT3B causes the reactivation of gene transcription through reversing CpG island methylation.

**Figure 11:**
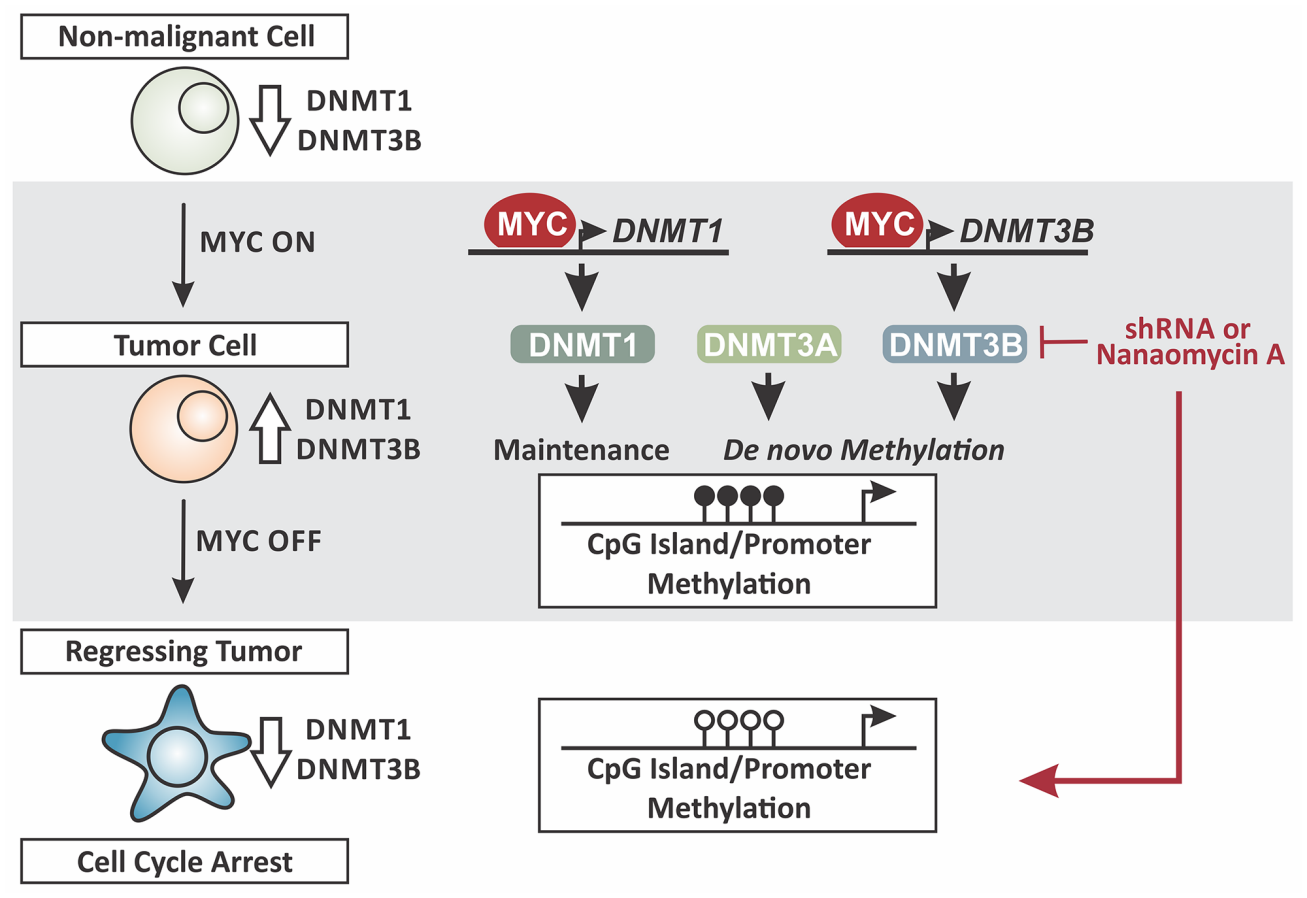
Working model MYC controls DNA methylation in a genome-wide fashion through overexpression of DNMT1 and DNMT3B. Non-malignant state: MYC levels are low, corresponding with low DNMT1 and DNMT3B expression. Tumor maintenance: MYC levels are constitutively high, driving the expression of DNMT1 and DNMT3B. Tumor regression/cell cycle arrest: MYC-inactivation in T-ALL causes tumor regression, associated with low DNMT1 and DNMT3B expression. Knock-down (shRNA) or pharmacologic inhibition (Nanaomycin A) of DNMT3B in tumor cells decreases proliferation. Loss of DNMT3B expression can reverse CpG island/promoter methylation thereby reactivating the corresponding genes. Taken together MYC induces and maintains global DNA methylation through control of a tumor cell-specific DNMT1 and DNMT3B expression.

Taken together, we propose a working model in which MYC, in addition to its role as a site-specific transcription factor, controls DNA methylation in a global manner through overexpression of DNMT1 and DNMT3B during tumor maintenance (Figure 11). In non-malignant cells MYC levels are low, corresponding with low DNMT1 and DNMT3B expression. This correlates with literature reporting hypomethylation of CpG islands and promoter regions of tumor suppressor genes, allowing for transcription with growth-limiting consequences [19–21]. In contrast, constitutively high MYC levels in tumor cells drive the expression of DNMT1 and DNMT3B. As consequence, tumor suppressor genes are silenced through hypermethylation in a genome-wide fashion. In parallel, DNMT3A is recruited by MYC-MIZ1 for repression of direct target genes via site-specific hypermethylation [18]. MYC inactivation in T-ALL causes tumor regression, which depends on reactivation of tumor suppressor genes that in turn trigger cellular senescence [6, 15, 22]. To allow their reactivation, CpG islands and promoter regions need to be hypomethylated, which is associated with diminished DNMT1 and DNMT3B levels.

Further expanding the list of chromatin modifying enzymes that MYC is known to regulate in the context of cancer, the direct control of the DNA methylating machinery reveals a novel facet of MYC’s global reach. It ties into the increasing number of reports that MYC acts as global regulator of chromatin structure (reviewed in [12]). The first chromatin modifier found to be directly deregulated by oncogenic MYC was the histone acetyltransferase *GCN5* which facilitates wide-spread acetylation of H3 and H4 [13]. More recently, MYC was also found to suppress chromatin regulatory genes such as *SIN3B*, *HBP1*, *SUV420H1* and *BTG1* through *miR-17-92* [14]. The epigenetic landscape that is established by the above direct MYC target genes then provides the framework for a secondary response from MYC as a global amplifier [54]. The latter model reveals that MYC boosts transcription through binding to euchromatic, active genes, while not being able to access heterochromatic regions. Finally, the ratio of MYC and MIZ1 occupying each promoter determines whether a gene is positively or negatively regulated [30, 55]. The control of genome-wide DNA methylation via DNMT1 and DNMT3B complements the model of MYC as a global regulator, allowing MYC to modulate the epigenetic landscape through DNA hypermethylation. Thereby it provides further evidence for the notion that both positive and negative transcriptional regulation is critical for MYC-driven tumor maintenance.

Further investigations are needed to unravel the complex interplay between the DNMT enzymes in establishing tumor-cell specific DNA methylation pattern. In the light of an elusive pharmacologic MYC inhibitor, our results highlight the importance of DNA methylation in MYC-driven tumor maintenance, and reveal the potential of specific components of the DNA methylating machinery for targeted therapeutic strategies.

## MATERIALS AND METHODS

### Ethics statement

Investigation has been conducted in accordance with the ethical standards and according to the Declaration of Helsinki and according to national and international guidelines and has been approved by the authors’ institutional review board.

### Cell culture and treatment

Mouse T-ALL cell lines were derived from the transgenic T-ALL mouse model (EμSRα-tTA;tet-o-MYC) [6]. Human Burkitt’s lymphoma-like cells (P493-6) [25], Burkitt’s lymphoma cell lines (Daudi, Raji and CA46) and T-cell leukemia/lymphoma cell lines (Jurkat, P12-Ichikawa, MOLT-4, CCRF-CEM and HPB-ALL) were obtained from ATCC. To turn off expression of the tetracycline-regulated MYC, 20 ng/mL doxycycline (DOX) was added to the cell culture medium for the indicated times; to titrate MYC expression levels, DOX was added at concentrations ranging from 0.1-0.5 ng/mL for 2 days. Cell lines were treated with 25nM, 50nM, and 100nM of the DNMT3B inhibitor Nanaomycin A which was obtained from Santa Cruz Biotechnology Inc. HEK293T cells were obtained from ATCC and maintained in DMEM supplemented with 10% FBS, 1% penicillin/streptomycin and 1% l-glutamine. All leukemia/lymphoma cell lines were passaged less than 8 times, maintained in RPMI1640 supplemented with 10% FBS, 1% penicillin/streptomycin, 1% l-glutamine and 50 μM 2-mercaptoethanol. Cell line viability with Trypan Blue was performed in triplicates using the Nexcelom Bioscience Cellometer2000Auto cell counter with Nexcelom Bioscience™ SD100 counting slides.

### shRNA-mediated knock-down

Cell lines were infected with lentiviral vectors (pTRIPZ or pLKO.1-puro) containing either scrambled control or specific shRNAs. Briefly, HEK293T cells were transfected using Lipofectamine 2000 (Invitrogen) with pTRIPZ/pLKO.1, pCMV-VSV-G and pCMV-dR8.2 dvpr plasmids. Virus particles were collected for spin infection. Upon selection of positive cells with 2-4 μg of puromycin, shRNA expression was induced with 100 ng/mL of DOX for the indicated times. The specific oligo sequences of shRNA are: MYC CCGGCCAAGGTAGTTATCCTTAAACTCGAGTTTAAGGATAACTACCTTGGTTTTTG; DNMT3B-sh1 TTGGCATTAGAATATCAGAGCCTCGAGGCTCTGATATTCTAATGCCAA; DNMT3B-sh2 AATTGCTGGGTACAACTTGGGCTCGAGCCCAAGTTGTACCCAGCAATT.

### Tissue

Human spleen (total RNA and protein lysate) obtained from a healthy donor, was purchased from Zyagen Inc. Human PBMCs (total RNA and protein lysate) and B-cells (total RNA) were obtained from the Augusta University Biorepository. 6-8 week old wild-type C57BL/6J mice were obtained from Jackson Laboratory. Mice were housed according to Augusta University’s animal care and IACUC guidelines.

### RNA extraction and analysis of gene expression

Total RNA was isolated using the NucleoSpin RNA Kit including DNase-I digest (Machery-Nagel Inc.) following the manufacturer’s protocol. 0.5 μg RNA were reverse transcribed into cDNA using the iScript cDNA Kit (BioRad). Quantitative PCR was performed using SYBR GREEN (BioRad) in an ABI StepOne Plus analyzer. The specific forward (F) and reverse (R) primer sequences are as follows: Hs *MYC* F: CTGCGACGAGGAGGAGAA, R: GGCAGCAGCTCGAATTTCTT; Hs *DNMT1* F: CC TAGCCCCAGGATTACAAGG, R: ACTCATCCGATT TGGCTCTTTC; Hs *DNMT3A* F: AGTACGACGA CGACGGCTA, R: CACACTCCACGCAAAAGCAC; Hs *DNMT3B* F: ACCTCGTGTGGGGAAAGATCA, R: CCA TCGCCAAACCACTGGA; Hs *ODC1* F: TTTACTG CCAAGGACATTCTGG, R: GGAGAGCTTTTAACCAC CTCAG; Hs *RPL13A* F: CGGACCGTGCGAGGTAT, R: CACCATCCGCTTTTTCTTGTC; Mm *MYC* F: TCTCC ATCCTATGTTGCGGTC, R: TCCAAGTAACTCGGTC ATCATCT; Mm *DNMT1* F: AAGAATGGTGTTGTC TACCGAC, R: CATCCAGGTTGCTCCCCTTG; Mm *DNMT3A* F: AAGGGGCCTTCAACGTG, R: ATTTATCC AGACTCGCGTGC; Mm *DNMT3B* F: GGCTT CAAGCCTACTGGGATCGAG, R: CCACAGGACAA ACAGCGGTCTTCC; Mm *ODC1* F: GACGAGTTTGA CTGCCACATC, R: CGCAACATAGAACGCATCCTT; Mm *SOX12* F: GGCTCCTCCCTAAGTCCATC, R: CC TAGCAACAACGTGCTTCA; Mm *FOXA1* F: TGGA CTTCAAGGCATACGAGC, R:GCACGGGTCTGGAAT ACACA; Mm *GLIS2* F: GACGAGCCCCTCGACCTAA, R: AGCTCTCGATGCAAAGCATGA; Mm *SPRY1* F: GGTCATAGGTCAGATCGGGTC, R: GTCCCGTATTC CACCATGCT; Mm *UBC* F: AGCCCAGTGTTACC ACCAAG, R: ACCCAAGAACAAGCACAAGG.

### Cell cycle analysis using propidium iodide

Cells were fixed in 70% methanol and stained using a Propidium Iodide (Acros Organics) solution containing PBS+0.5% BSA, 50μg/mL PI, and 200μg/mL RNaseA. Cells were then analyzed immediately on a LSR II flow cytometer (Becton Dickinson). FACS data was analyzed using FlowJo software (Tree Star).

### Flow cytometric analysis of cell death

Annexin V and propidium iodide staining was used for the study of cell cycle distribution and apoptosis using the Annexin V-FITC Early Apoptosis Detection Kit from Cell Signaling. Briefly, cells were resuspended in PBS and fixed with ice-cold ethanol. Cells were treated with RNase and propidium iodide and analyzed on a LSR II flow cytometer (Becton Dickinson). FACS data was analyzed using FlowJo software (Tree Star).

### Statistical analysis

All experiments were performed on biological replicates unless otherwise specified. Sample size is reported in the respective figure legends. All quantitative PCR were run in triplicates and standard deviation is shown in the figures. Two-tailed Student’s *t*-test was used to calculate *p*-values; statistically significant values are specified in the figure legends.

### Cell extracts and western blot analysis

Total protein extracts were prepared using a lysis buffer (50 mM Tris, 2% SDS, 10% glycerol, 0.74 M beta-mercaptoethanol), sonicated on ice using a Sonifier 250D (Branson Ultrasonics), and heated for 5 min at 99 °C. Protein concentrations were determined with the Bio-Rad DC Protein Assay Kit (BioRad) using bovine serum albumin as a standard. Protein extracts were separated in 10% SDS-PAGE and electrotransferred to PVDF membranes (Immobilon-P; EMD Millipore). Antibodies used were as follows: MYC #ab32 (Abcam, Cambridge, USA) [56], #sc764 (Santa Cruz Biotechnology, USA) [57]; DNMT3B #ab122932 (Abcam, Cambridge, USA) [58],#nb100-56514 (Novus Biologicals, USA) [59]; TUBULIN #ab6046 (Abcam, Cambridge, USA) [22]; β-ACTIN #A5441 (Sigma-Aldrich, USA); GAPDH #sc25778 (Santa Cruz Biotechnology, USA) [60]; Anti-MOUSE HRP #31430 (Thermo Fisher, USA) [61]; Anti-RABBIT HRP #31460 (Thermo Fisher, USA) [62]. Protein quantitation was performed using ImageJ software (https://imagej.nih.gov/ij/) [63].

### Chromatin immunoprecipitation (ChIP) analysis

For ChIP, cells were crosslinked with 1% formaldehyde followed by quenching with 0.2 M glycine. After washing with PBS, the cells were resuspended in lysis buffer (10 mM EDTA pH 8.0, 50 mM Tris-HCl pH 8.0, 1% SDS). Chomatin was sheared by sonication for 10 min using a Branson 250 Sonifier, diluted in ChIP Dilution Buffer (0.01% SDS, 1.1% Triton X-100, 1.2 mM EDTA pH 8.0, 16.7 mM Tris-HCl pH 8.0 and 167 mM NaCl) and incubated with 5 μg of specific antibody overnight. The bound material was recovered after 2 hours incubation with 50 μl Dynabeads/Protein G (Invitrogen/Thermo-Fisher), rotating at 4°C. The beads were washed, once in Low Salt Buffer (0.1% SDS, 1% Triton X-100, 2 mM EDTA pH 8.0, 20 mM Tris-HCl pH 8.0 and 150 mM NaCl), twice in High Salt Buffer (0.1% SDS, 1% Triton X-100, 2 mM EDTA pH 8.0, 20 mM Tris-HCl pH 8.0 and 500 mM NaCl), twice in LiCl Buffer (0.25 M LiCl, 1% NP-40, 1% Na-Deoxycholate, 1 mM EDTA pH 8.0 and 10 mM Tris-HCl pH 8) and twice in TE buffer. ChIPed material was eluted by two 15 minute incubations at room temperature with 250 μl Elution Buffer (1% SDS and 0.1 M NaHCO_3_). Chromatin was reverse-crosslinked by adding 0.2 M NaCl for 4 hours at 65°C. After RNase and proteinase K treatment, DNA was extracted using phenol-chloroform.

ChIP followed by promoter array analysis (ChIP-chip) was performed using the 244K Mouse Promoter Microarray Kit (Agilent Technologies) covering ~19,000 defined mouse genes following the manufacturer’s protocol. ChIP DNA was used for microarray hybridization following the manufacturer’s protocol. ChIP-chip data processing was performed using Agilent Technologies ChIP Analytics Platform v1.3.1 by mapping to data to NCBI36/mm8. ChIP figures were generated using the UCSC genome browser (https://genome.ucsc.edu/). E-box motif search was performed for the genomic DNA sequence surrounding MYC binding peaks using JASPAR (http://jaspar.genereg.net) [31].

ChIP followed by quantitative PCR was performed using SYBR GREEN (BioRad) in an ABI StepOne Plus analyzer. The specific forward (F) and reverse (R) primer sequences and their genomic location are as follows: Mm *DNMT1* ChIP-1 (chr9:20,700,891-20,701,004) F: CATGGCCACAACATCTCACT, R: TTATCTCACCA GCCCCAGAC; Mm *DNMT1* ChIP-2 (chr9:20,704,688-20,704,791) F: GAATGACCGAGGACCAGAAA, R: TG TGTACATGTGCGTGGGTA; Mm *DNMT1* ChIP-3 (chr9:20,706,395-20,706,510) F: TCTTAGTAGCCAGG GCCAGA, R: TGGGTGCAGCTCTGAGTATG; Mm *DNMT3B* ChIP-1 (chr2:153,340,243-153,340,357) F: TT GTGTTTCTCCAGTGGTTCAG, R: CATACATGTTCC CCCAACTACC; Mm *DNMT3B* ChIP-2 (chr2:153, 342,147-153,342,265) F: CTCAGTAGAGTGCTTCC GGACT, R: TACCTGAGGTTTCCAAGGTCTG; Mm *DNMT3B* ChIP-3 (chr2:153,343,559-153,343,676) F: AGA TAGCGCTTGCTAAATCTGG, R: CACTACCTGGGGG TAAAGAACA;

### Reduced representation bisulfite sequencing (RRBS)

RRBS was performed by Diagenode, Inc. as described before [64]. Sequencing was carried out on an Illumina HiSeq 3000. Quality control of sequencing reads was performed using FastQC [65]. Adapter removal was performed using Trim Galore! v0.4.1 [66]). Reads were then aligned to the reference genome using bismark v0.16.1 [67], followed by methylation calling using the corresponding bismark functionality. The comparative RRBS analysis was carried out using methylKit [68], based on reference genome mm9 and CpG island annotation from UCSC [69]. Differentially methylated CpGs, as well as differentially methylated regions (DMRs) were identified (the latter with a window size of 1,000 bp, as this has been found to include the majority of DMRs [70]). RRBS data has been deposited under GEO accession number GSE101907 (https://www.ncbi.nlm.nih.gov/gds).

## SUPPLEMENTARY MATERIALS FIGURES AND TABLES



## Abbreviations

BTG1: B-Cell translocation gene 1
CDKN: Cyclin dependent kinase inhibitor
GAPDH: Glyceraldehyde 3-phosphate dehydrogenase
GCN5: Lysine acetyltransferase 2A
HPB1: Human polybromo-1 protein
Hs: Human
MIZ-1: Myc-interacting zinc finger protein 1
Mm: Mouse
MYC: Avian myelocytomatosis viral oncogene homolog
NCL: Nucleolin
RPL13A: Ribosomal protein L13a
SIN3B: Histone deacetylase complex subunit Sin3b
SUV420H: Suppressor of variegation 4-20 homolog
UBC: Ubiquitin
