# Supplementary material for: DNMT3B overexpression contributes to aberrant DNA methylation and MYC-driven tumor maintenance in T-ALL and Burkitt’s lymphoma
Source: Oncotarget. 2017 Aug 10;8(44):76898–920. doi: 10.18632/oncotarget.20176 (PMC5652751; doi:10.18632/oncotarget.20176)
Supplement: Supplementary file 1 [file oncotarget-08-76898-s001.pdf]

DNMT3B overexpression contributes to aberrant DNA methylation and MYC-driven tumor maintenance in T-ALL and Burkitt's lymphoma

SUPPLEMENTARY MATERIALS

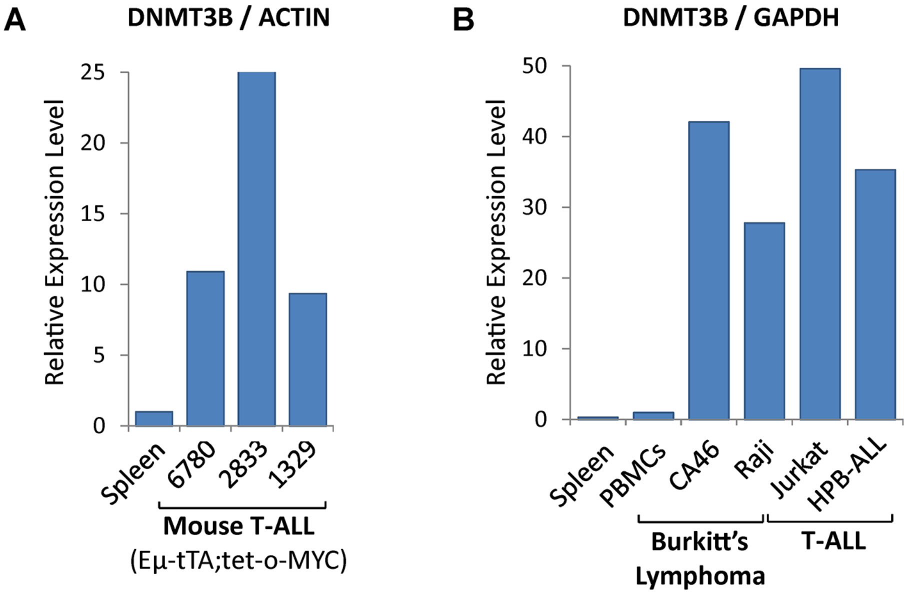

Supplementary Figure 1: Quantitation of DNMT3B Western blot analysis in T-ALL and Burkitt's lymphoma cell lines. Protein quantitation of DNMT3B Western blot results displayed in Figure 1B and E using ImageJ software. **(A)** T-ALL cell lines (6780, 2833 and 1329) derived from a transgenic T-ALL mouse model (EμSRα-tTA;tet-o-MYC) in comparison to spleen tissue obtained from wild-type C57BL/6J mice. **(B)** Human T-ALL (Jurkat and HPB-ALL) and Burkitt's lymphoma (CA46 and Raji) compared to normal spleen, peripheral blood mononuclear cells (PBMCs) and B-cells obtained from healthy donors. Data was normalized to ACTIN or GAPDH.

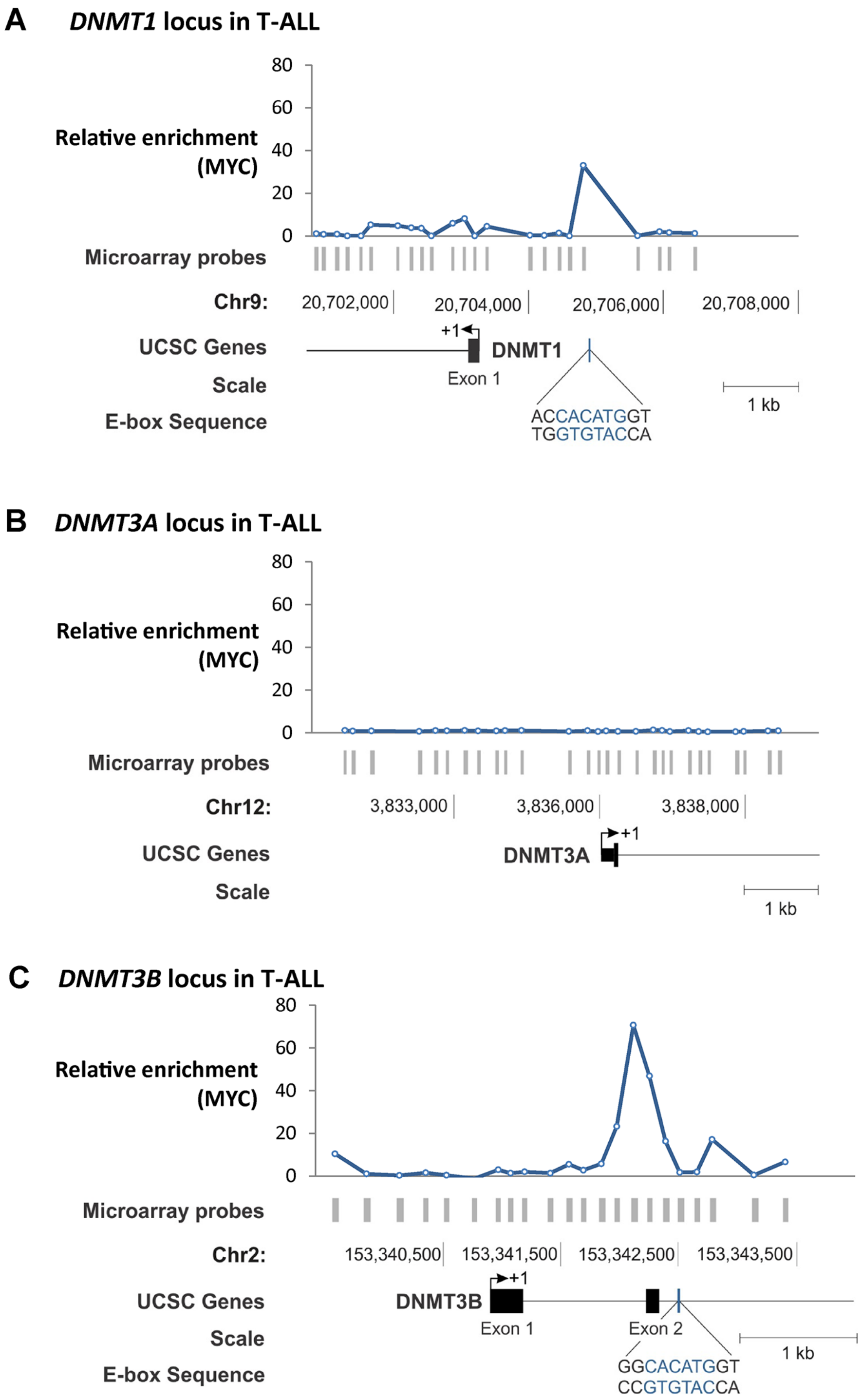

Supplementary Figure 2: ChIP-chip analysis for MYC binding to the *DNMT1*, *DNMT3A* and *DNMT3B* loci in T-ALL cells. Agilent mouse promoter microarrays covering -5.5kb to +2.5kb of the transcription start site were used for ChIP-chip analysis. ChIP-chip data (IP: MYC) for mouse T-ALL cells (EμSRα-tTA;tet-o-MYC) indicating the relative enrichment (Y axis) for the genomic loci (X axis) of **(A)** *DNMT1* **(B)** *DNMT3A* and **(C)** *DNMT3B*. The chromosomal location is indicated in bp, and the scale in kb. The location of the microarray probes is indicated by grey vertical bars. E-box sequences are shown for the vicinity of MYC binding peaks. Exons are displayed as black vertical bars, the UTR is represented by a line, and the transcription start site (TSS) is marked by an arrow indicating the direction of transcription. E-box sequences are shown for the vicinity of MYC binding peaks. Schematic was generated using the UCSC Genome Browser.

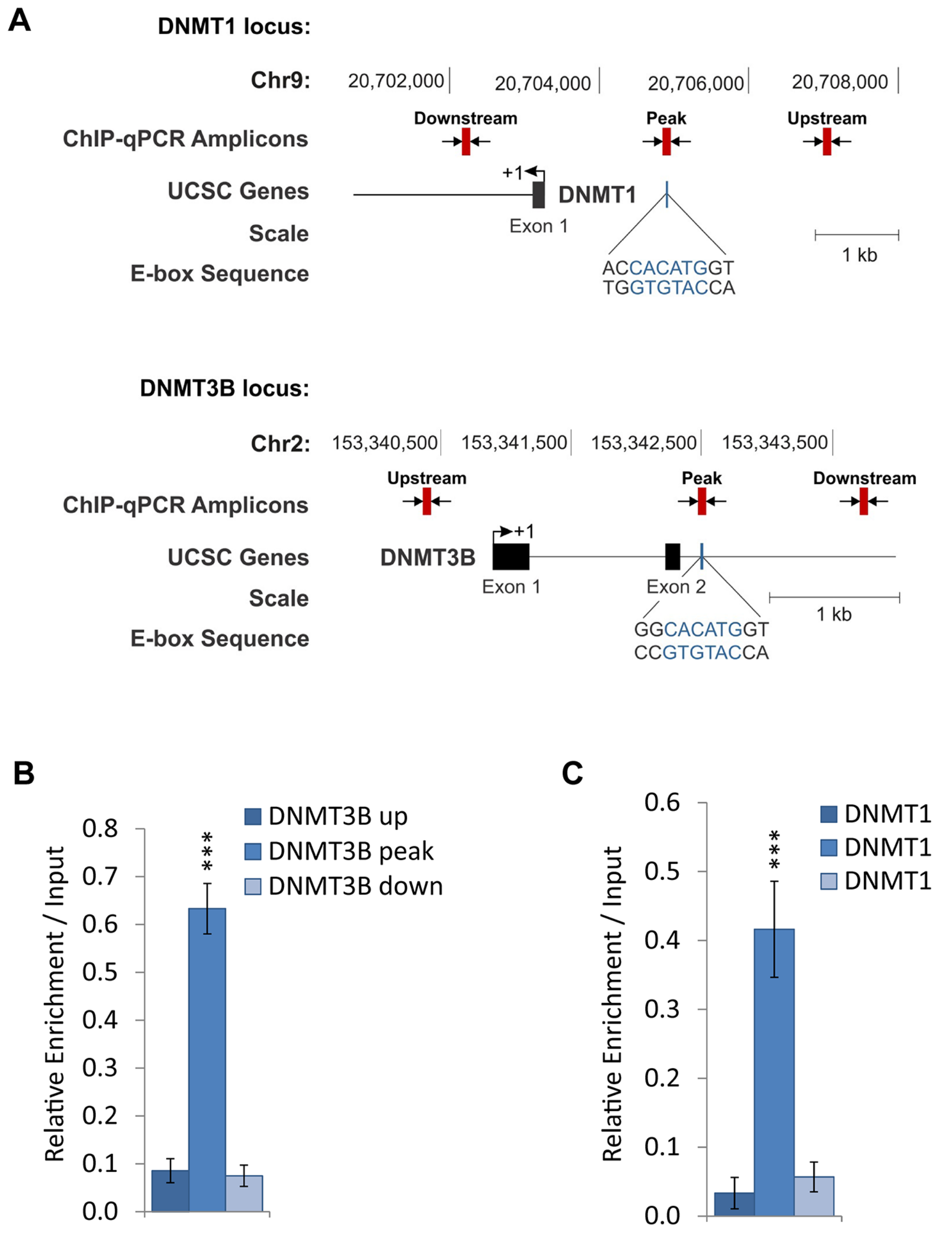

Supplementary Figure 3: ChIP-qPCR validation of MYC binding to the *DNMT1* and *DNMT3B* loci. **(A)** Schematic of amplicon locations used for ChIP-qPCR validations. ChIP-qPCR for **(B)** *DNMT1* and **(C)** *DNMT3B* showing MYC or IgG enrichment (ChIP/input) for peak (peak), downstream (down) and upstream (up) regions in mouse T-ALL (EμSRα-tTA;tet-o-MYC) cells. Error bars represent mean ± SEM; $n = 3$; two-tailed Student's t-test: $^{***}P < 0.001$.

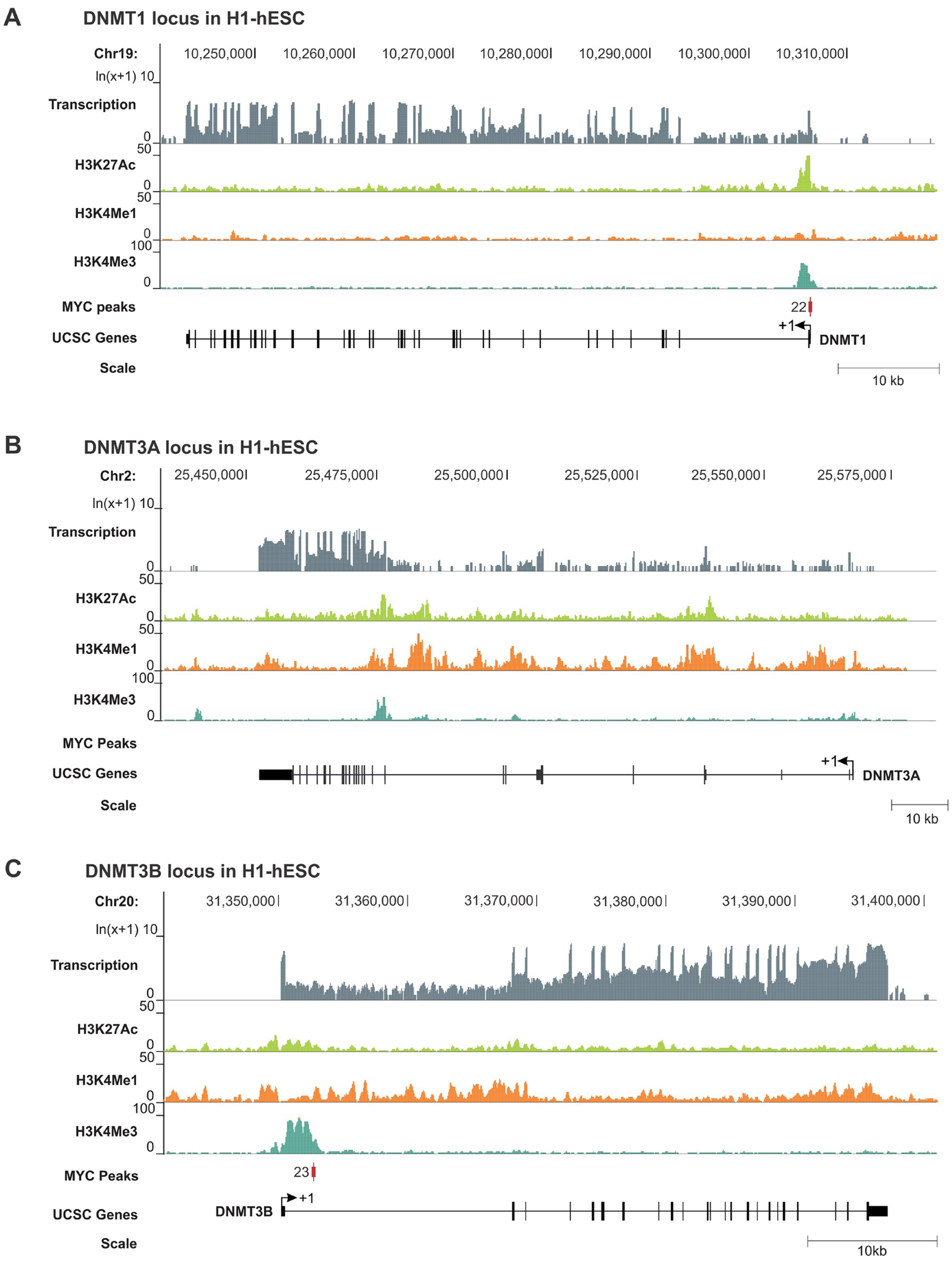

Supplementary Figure 4: RNA-seq and ChIP-seq analysis for H3K27Ac, H3K4Me1, H3K4Me3 and MYC for the *DNMT1*, *DNMT3A* and *DNMT3B* loci in human embryonic stem cells. Publically available RNA-seq (transcription) and ChIP-seq data (IP: H3K27Ac, H3K4Me1, H3K4Me3 and MYC) for human embryonic stem cells (H1-hESCs) were analyzed to display the relative enrichment (Y axis) for the genomic loci (X axis) of **(A)** *DNMT1* **(B)** *DNMT3A* and **(C)** *DNMT3B*. ENCODE [32] data sets: RNA-seq (GSM958737), H3K27Ac (GSM733718), H3K4Me1 (GSM733782), H3K4Me3 (GSM733657), MYC (GSM935509). Enrichment peaks for MYC are displayed as red vertical bars; the number represents the signal value. The chromosomal location is indicated in bp, and the scale in kb. Exons are displayed as black vertical bars, the UTR is represented by a line, and the transcription start site (TSS) is marked by an arrow indicating the direction of transcription. Schematic was generated on reference genome hg19 using the UCSC Genome Browser.

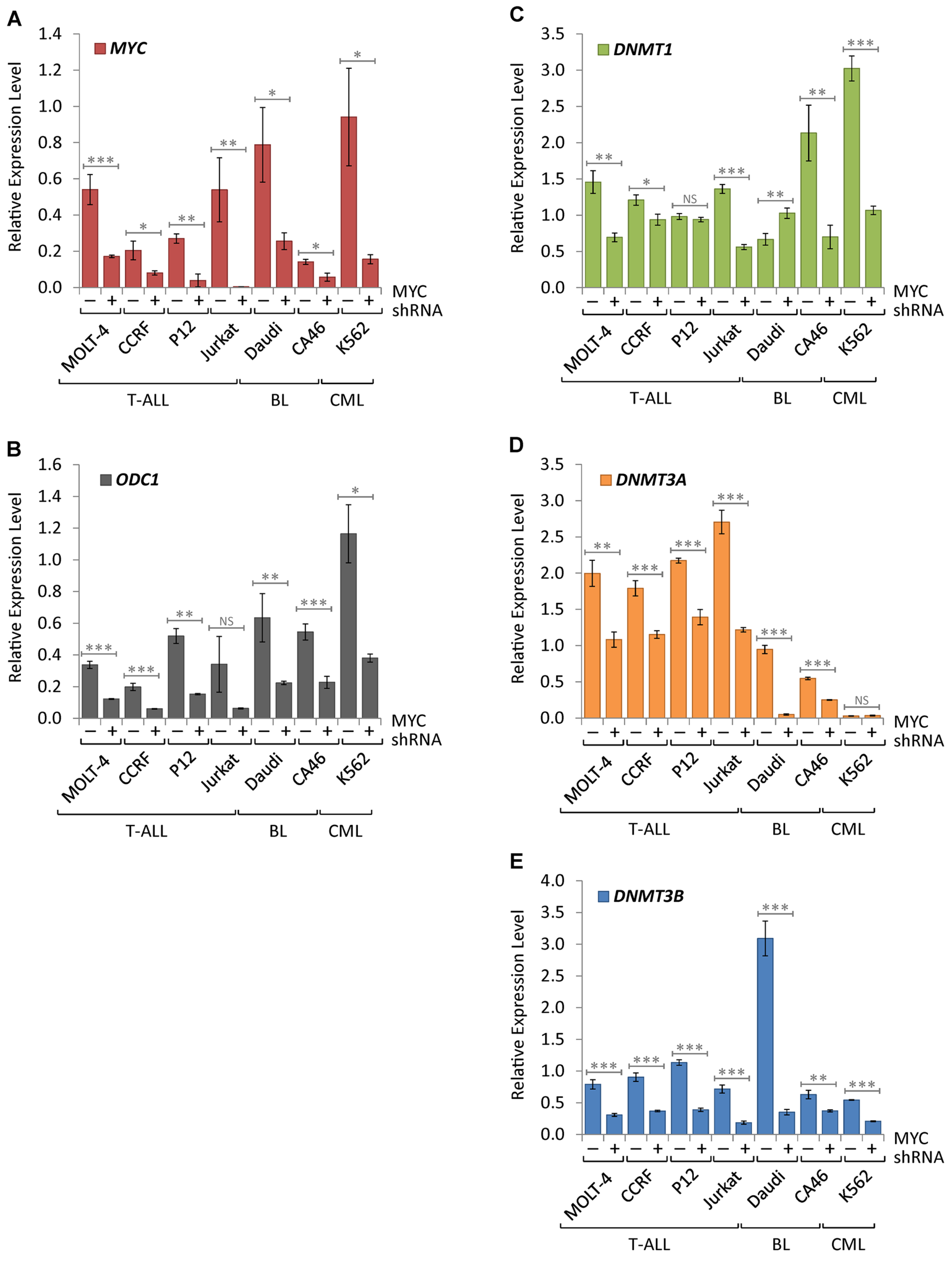

Supplementary Figure 5: Knockdown of endogenous *MYC* leads to diminished *DNMT3B* expression levels in human T-ALL and Burkitt's lymphoma cell lines. shRNA-mediated knockdown of endogenous *MYC* in human T-ALL and Burkitt's lymphoma cell lines. RT-qPCR analysis of **(A)** *MYC* and **(B)** its canonical target gene, *ODC1*, serving as control, as well as **(C)** *DNMT1*, **(D)** *DNMT3A* and **(E)** *DNMT3B*. Human T-ALL (MOLT-4, CCRF-CEM, P12-Ichikawa and Jurkat), human Burkitt's lymphoma (Daudi and CA46) cell lines before and upon tetracycline-inducible knock-down of MYC (+MYC shRNA) for 2 days. RT-qPCR data was normalized to UBC. Error bars represent mean ± SEM; $n = 3$; two-tailed Student's t-test: NS = non-significant; *$P < 0.05$; **$P < 0.01$; ***$P < 0.001$.

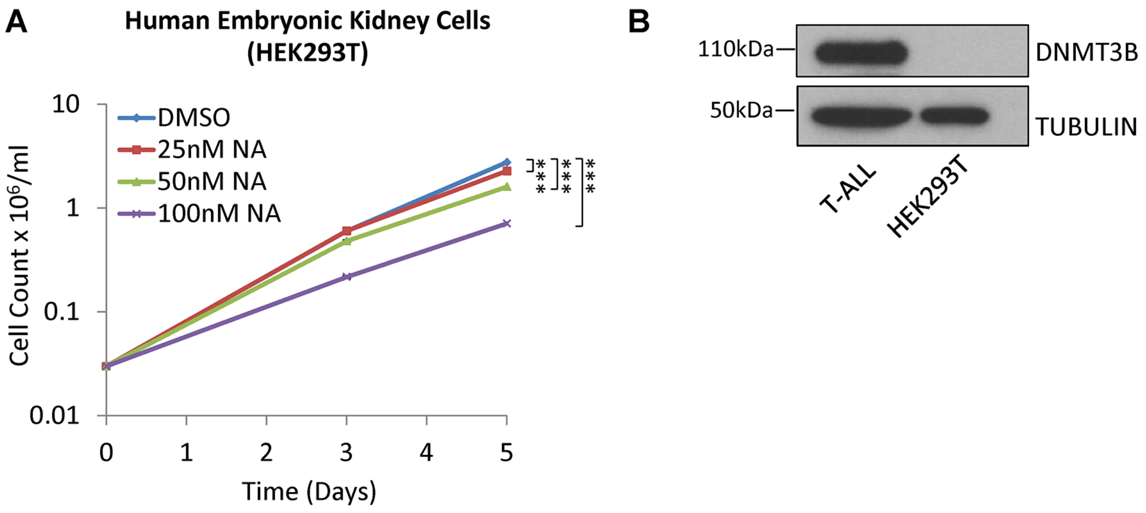

Supplementary Figure 6: Pharmacologic inhibition of DNMT3B in non-malignant HEK293T cells. Human embryonic kidney cells (HEK293T) were treated with 25nM, 50nM, and 100nM of the DNMT3B inhibitor Nanaomycin A, compared to DMSO control. **(A)** Growth curve comparing viable cell counts plotted on a logarithmic scale. Error bars represent mean ± SEM; $n = 3$; two-tailed Student's t-test: ***$P < 0.001$. **(B)** Western blot expression analysis of DNMT3B protein in T-ALL (6780) and HEK293T cells.

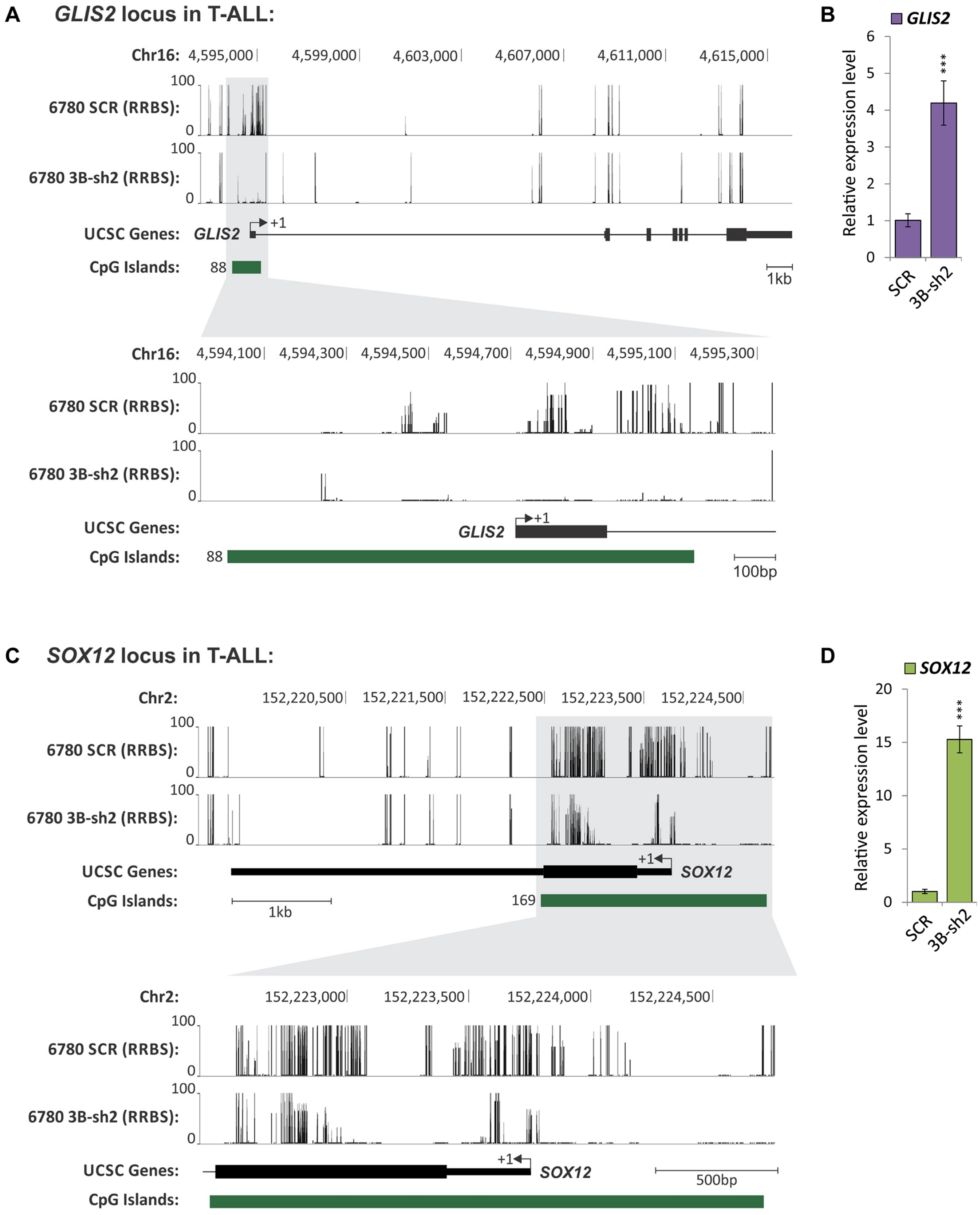

Supplementary Figure 7: Gene-specific DNA methylation analysis of T-ALL before and upon DNMT3B knock-down using RRBS. T-ALL (EμSRα-tTA;tet-o-MYC) cells were analyzed upon shRNA-mediated knock-down of DNMT3B (6780 3B-sh2) compared to scrambled control cells (6780 SCR) by RRBS. DNA methylation is displayed for the genomic loci of **(A)** *SOX12* (*SRY (sex determining region Y)-box 12*), and **(C)** *GLIS2* (*GLIS family zinc finger 2*). Methylation level of CpG nucleotides is indicated on the Y axis; genomic location is indicated on the X axis. The chromosomal location is indicated in bp, and the scale in kb. Exons are displayed as black vertical bars, the UTR is represented by a line, and the transcription start site (TSS) is marked by an arrow indicating the direction of transcription. CpG islands are displayed as green bars; the number of CpGs per island is indicated. Schematic was generated based on reference genome mm9 using the UCSC Genome Browser. RT-qPCR expression profiling of **(B)** *SOX12*, and **(D)** *GLIS2* before (SCR) and upon knockdown of DNMT3B (3B-sh2) in T-ALL (6780) cells. RT-qPCR was normalized to *UBC*. Error bars represent mean ± SEM; n = 3; two-tailed Student's t-test: ***$P < 0.001$.
